# The HCMV gH/gL/UL128-131 Complex Triggers the Specific Cellular Activation Required for Efficient Viral Internalization into Target Monocytes

**DOI:** 10.1371/journal.ppat.1003463

**Published:** 2013-07-11

**Authors:** Maciej T. Nogalski, Gary C. T. Chan, Emily V. Stevenson, Donna K. Collins-McMillen, Andrew D. Yurochko

**Affiliations:** 1 Department of Microbiology and Immunology, Center for Molecular and Tumor Virology, Louisiana State University Health Sciences Center, Shreveport, Louisiana, United States of America; 2 Feist-Weiller Cancer Center, Louisiana State University Health Sciences Center, Shreveport, Louisiana, United States of America; Oregon Health and Science University, United States of America

## Abstract

We have established that HCMV acts as a specific ligand engaging and activating cellular integrins on monocytes. As a result, integrin signaling *via* Src activation leads to the functional activation of paxillin required for efficient viral entry and for the biological changes in monocytes needed for viral dissemination. These biological/molecular changes allow HCMV to use monocytes as “vehicles” for systemic spread and the establishment of lifelong persistence. However, it remains unresolved how HCMV specifically induces this observed monocyte activation. It was previously demonstrated that the HCMV gH/gL/UL128-131 glycoprotein complex facilitates viral entry into biologically relevant cell types. Nevertheless, the mechanism by which the gH/gL/UL128-131 complex promotes this process is unknown. We now show that only HCMV virions possessing the gH/gL/UL128-131 complex are capable of activating integrin/Src/paxillin-signaling in monocytes. In fibroblasts, this signaling is reversed, such that virus lacking the gH/gL/UL128-131 complex is the only virus able to induce the paxillin activation cascade. The presence of the gH/gL/UL128-131 complex also may have an inhibitory effect on integrin-mediated signaling pathway in fibroblasts. Furthermore, we demonstrate that the presence of the gH/gL/UL128-131 complex on the viral envelope, through its activation of the integrin/Src/paxillin pathway, is necessary for efficient HCMV internalization into monocytes and that appropriate actin and dynamin regulation is critical for this entry process. Importantly, productive infection in monocyte-derived macrophages was seen only in cells exposed to HCMV expressing the gH/gL/UL128-131 complex. From our data, the HCMV gH/gL/U128-131 complex emerges as the specific ligand driving the activation of the receptor-mediated signaling required for the regulation of the actin cytoskeleton and, consequently, for efficient and productive internalization of HCMV into monocytes. To our knowledge, our studies demonstrate a possible molecular mechanism for why the gH/gL/UL128-131 complex dictates HCMV tropism and why the complex is lost as clinical isolates are passaged in the laboratory.

## Author Summary

We previously demonstrated that HCMV, by engaging cellular receptors, changes the biology of blood monocytes, allowing for efficient viral entry into these cells and their use as virus-carriers in HCMV systemic spread. However, it was unclear how HCMV induces receptor-mediated signaling in infected cells. Here we report that HCMV by expressing a specific complex of five glycoproteins, present on HCMV clinical isolates, engages cellular integrin receptors and subsequently triggers integrin-mediated signaling leading to efficient viral entry into monocytes and productive infection of monocyte-derived macrophages. We also demonstrate that the HCMV pentameric complex has an inhibitory effect on integrin-mediated signaling in fibroblasts, an *in vitro* model system of HCMV infection, suggesting that the presence of the pentameric complex is not advantageous for HCMV infection of fibroblasts. Together, our results argue that HCMV uses distinct mechanisms to enter monocytes and fibroblasts. In support, our findings indicate that HCMV utilizes an endocytic-like route of entry into monocytes that is in contrast to viral fusion at the cell surface seen in fibroblasts. Our studies provide a molecular explanation for a previously observed critical role of the HCMV pentameric complex during infection of clinically relevant cell types, which in the future may lead to the development of better targets for antiviral therapy.

## Introduction

Human cytomegalovirus (HCMV) is a betaherpesvirus characterized by worldwide prevalence in the human population. Although infection of immunocompetent individuals is usually mild or asymptomatic, increasing evidence shows that HCMV infection is a strong risk factor in the development of several cardiovascular diseases (CVDs) [1]–[5], and that the infection may lead to the development of some cancers [6], [7]. In immunocompromised individuals, viral infection can lead to significant morbidity and mortality [8], [9]. HCMV is the leading viral cause of congenital central nervous system damage and a leading opportunistic pathogen in AIDS and transplant patients [8], [9]. The virus is shed in nearly all body fluids illustrating HCMV's broad cellular tropism and capacity to spread to and infect most organ systems. It is this broad tropism and multiple organ system involvement that lead, in susceptible individuals, to the hallmark of HCMV pathogenesis - multiorgan failure [9]–[15]. It is thought that, for HCMV to cause broad-organ pathogenesis, infected circulating cells in the blood act as viral-carriers allowing for dissemination of the virus to multiple target tissues. In support, HCMV infection is characterized by a cell-associated viremia, in particular a monocyte-associated viremia prior to the onset of viral pathogenesis [9], [15]–[18]. As a cell type, monocytes are characterized by high motility and the capacity to migrate to all host organ systems making them an ideal cell type for viral dissemination [19]–[21].

We have previously shown that HCMV infection of monocytes leads to a wide range of biological changes that shape the behavior of target monocytes. HCMV-infected monocytes are characterized by the overexpression and secretion of inflammatory cytokines, an enhanced cellular motility, the increased expression of adhesion molecules allowing for tight adhesion of infected monocytes to endothelial cells, an increase in transendothelial migration, and the promotion of cellular differentiation [16]–[18]. Importantly, monocytes are not permissive for HCMV gene expression and replication upon initial infection and have to differentiate into monocyte-derived macrophages to support productive infection [17], [22]–[24]. This wide range of molecular changes in monocytes during HCMV infection and lack of productive infection in HCMV-infected monocytes sets this cell type apart from other cell types, underlying the unique biological processes hijacked by HCMV during infection of monocytes.

Molecular changes in monocytes begin to occur within minutes post infection, suggesting that a receptor/ligand process initiates changes during the early steps of HCMV infection [16], [17]. In support, UV-irradiated, virus-treated monocytes showed the same changes in transcription factor regulation, cellular signaling and motility as “live” virus [16]–[18]. Additionally, studies demonstrated that HCMV glycoproteins gB and gH also induced rapid activation of Specificity Protein 1 (Sp1) and nuclear factor κ-light-chain-enhancer of activated B cells (NF-κB) transcription factors [16], [25], indicating that the act of HCMV binding to target cells triggers biological changes. Recently, we demonstrated that HCMV engages the epidermal growth factor receptor (EGFR), and the β1 and β3 integrins on the surface of monocytes to initiate the receptor-mediated signaling pathways found to be critical for efficient HCMV internalization and virus-induced “hyper” cellular motility [26], [27]. Furthermore, our results showed that HCMV engagement of each individual receptor (EGFR *vs.* β-integrins) initiated specific changes in HCMV-infected monocytes, such that viral binding of each receptor directed distinct events that modulated viral entry and pathogenic cellular motility [22], [26], [27]. Specifically for β1 and β3 integrins, HCMV engagement triggers the activation of the Src/paxillin-signaling axis [27]. Thus, HCMV appears to utilize receptor-initiated signal transduction pathways in monocytes to shape the biology of these cells despite the initial lack of viral gene expression and replication.

We propose that the ability of HCMV to induce distinct combinations of signal transduction pathways resulting in HCMV-specific functional changes in monocytes is determined by the nature of the viral glycoproteins expressed on the mature viral envelope. HCMV possesses several major envelope glycoprotein complexes with the glycoprotein B (gB) and glycoprotein H (gH) complexes being the apparent dominant signaling complexes shown to interact with their cognate cellular receptors, EGFR and β-integrins, respectively [28], [29]. HCMV gB may also interact with integrins on fibroblasts [29]. Nevertheless, at least on fibroblasts, it appears that the gH complex is the dominant glycoprotein complex responsible for binding to integrins - a receptor-ligand engagement implicated in HCMV entry [28]. With our new data showing that appropriate integrin signaling is required for efficient HCMV entry into blood monocytes, we have now focused our studies on understanding the biological consequences of the gH-integrin interaction. HCMV expresses several types of gH complexes: a dimeric gH/gL complex, a trimeric gH/gL/gO complex, which is sufficient for attachment to and infection of fibroblasts [30], [31], and a multimeric gH/gL/UL128-131 complex, which is required for infection of dendritic, endothelial, and epithelial cells [31]–[34], virus transfer to leukocytes [30] and, according to a recent report, the infection of monocytes [35]. The gH/gL/UL128-131 complex is made of five proteins: gH, gL, the UL128 protein (pUL128), pUL130 and pUL131. It is only found expressed on clinical strains of HCMV and is not present or is non-functional in laboratory-adapted strains (i.e. AD169) that have been extensively cultured in fibroblasts [30], [36]–[39]. Moreover, coding sequences of the UL128, UL130 and UL131A genes are conserved between clinical isolates, suggesting the importance these three proteins play during *in vivo* infection [40]. Nevertheless, the mechanism by which the gH/gL/UL128-131 complex promotes viral attachment and entry is unknown. It has been argued that the presence or absence of the UL128-131 complex dictates tropism due to the capacity of this complex to bind to different cell types [41]. We postulate that this idea is an oversimplification of the role the UL128-131 complex plays during infection; that is, this region not only dictates binding, but it also dictates the type and/or levels of receptor-mediated signaling in target cells.

To investigate the role the gH/gL/UL128-131 complex plays in the ability of HCMV to induce signal transduction pathways in target monocytes, we used an AD169 clone (BAD*wt*) produced from a bacterial artificial chromosome (BAC), containing a frameshift mutation in UL131A, and the virus (BAD*r*UL131) containing a repaired and functional gH/gL/UL128-131 complex [31]. Here, we demonstrated that in monocytes only BAD*r*UL131 is able to induce the integrin/Src/paxillin-signaling pathway – a signaling axis we previously showed to be critical for efficient HCMV entry into and enhanced motility of target monocytes [27]. We also show in this current report that this signaling is reversed in fibroblasts, such that BAD*wt* is the only virus able to induce the paxillin activation cascade. In addition, the presence of the gH/gL/UL128-131 complex appears to have an inhibitory effect on integrin-mediated signaling in fibroblasts. Furthermore, our studies reveal that the presence of the gH/gL/UL128-131 complex on the viral envelope, through its activation of the integrin/Src/paxillin pathway, is necessary for efficient HCMV internalization into monocytes and productive infection in monocyte-derived macrophages. Additionally, the entry efficiency of these viruses (with and without the UL128-131 complex) was unchanged during infection of wild type or paxillin-deficient fibroblasts, strongly suggesting contrasting mechanisms of entry into monocytes *vs.* fibroblasts. Our results indicate that the gH/gL/UL128-131 complex promotes viral internalization through the regulation of actin rearrangement and dynamin, suggesting a macropinocytosis-like route of entry into target monocytes [42]–[44]. From our data, the HCMV gH/gL/U128-131 complex emerges as the specific ligand that is necessary for the activation of the receptor-mediated signaling pathways required for the regulation of the actin cytoskeleton and, consequently, for efficient and productive internalization of HCMV into monocytes. Together, our new studies are the first to document a possible molecular mechanism for why the gH/gL/UL128-131 complex dictates HCMV tropism and why there is a selective pressure to lose this complex as clinical isolates are passaged in the laboratory.

## Results

### Expression of the gH/gL/UL128-131 complex on HCMV strains links the activation of the integrin/Src-mediated signaling pathway to efficient viral internalization into target monocytes

**Figure 1:**
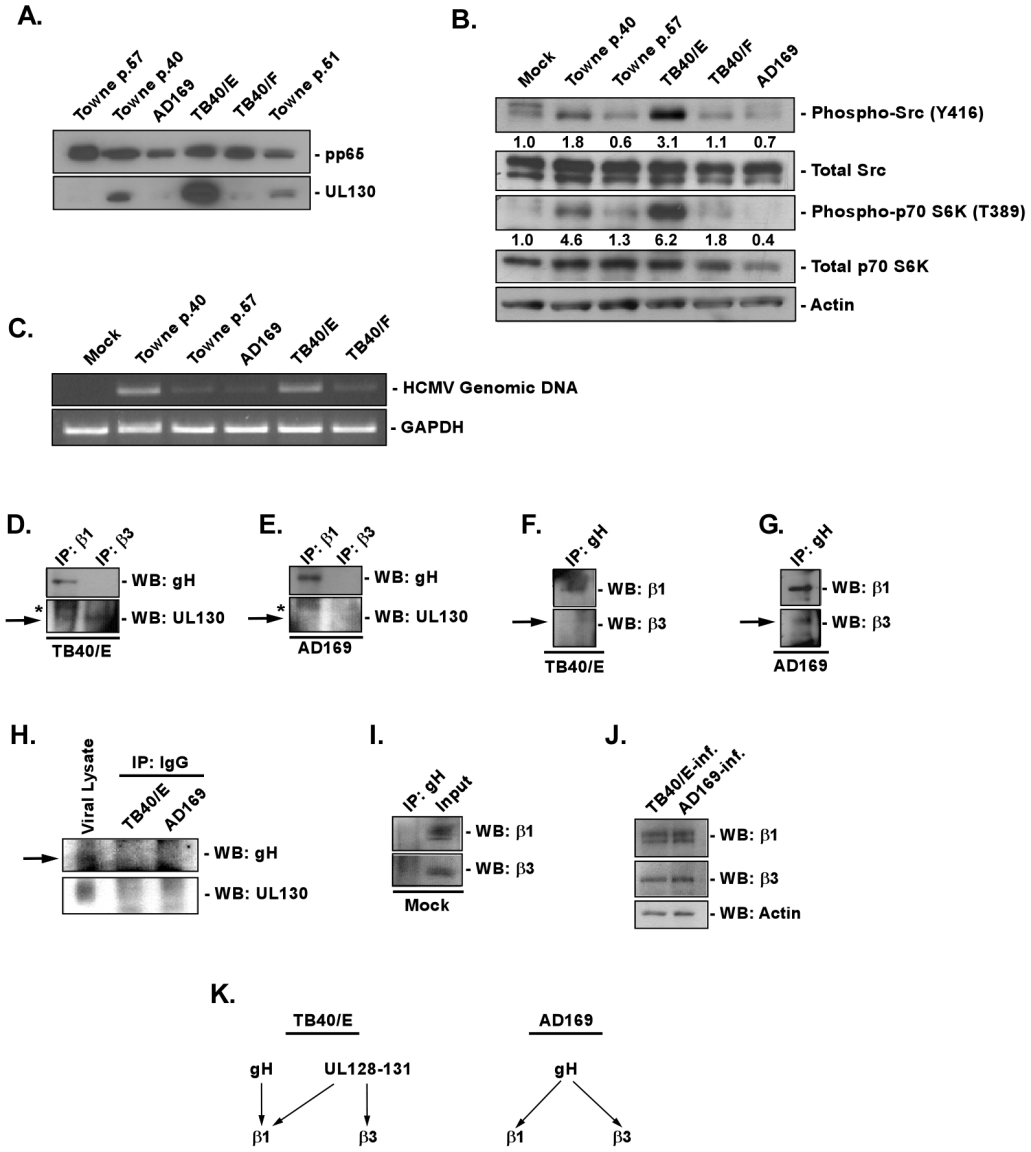
Presence of the gH/gL/UL128-131 complex links HCMV′s ability to activate and efficiently enter into target monocytes. (A) Approximately 2×10^6^ virions of Towne (p.40, p.51 and p.57), AD169, TB40/E and TB40/F were spun down through a sucrose cushion, lysed and western blot analyses were performed using antibodies recognizing the HCMV proteins, pp65 and pUL130. (B) Monocytes were isolated and cultured in low serum for 24 h at 37°C/5% CO_2_. Monocytes were then mock- or HCMV (Towne p.40, Towne p.57, TB40/E, TB40/F, AD169)-infected (M.O.I. of 5) and harvested at 15 min. pi. Western blot analyses were performed using antibodies specific for the phosphorylated and non-phosphorylated forms of Src and p70 S6 kinase. Actin was used as a loading control. The results were also measured by densitometry with relative numbers shown in the figure. (C) Monocytes were mock infected or HCMV (Towne p.40, Towne p.57, TB40/E, TB40/F, AD169) infected (M.O.I. of 0.1) for 1 h at 4°C, then temperature shifted to 37°C for 1 h. Monocytes were washed and treated with Proteinase K solution for 1 h. Monocytes were then harvested and semi-quantitative PCR was performed using primers complementary to genomic HCMV DNA and cellular GAPDH, as an internal control. PCR reactions were analyzed by agarose gel electrophoresis using ethidium bromide. (D, E, F, G, H, I and J) Monocytes were mock- or HCMV (TB40/E or AD169)-infected (M.O.I. of 5) for 1 h at 4°C, then 2 mM of DTSSP [3,3′-dithiobis (sulfosuccinimidylpropionate] was added at 4°C for additional 2 h. Cells were spun down and lysed. Antibodies recognizing β1, β3 integrins, HCMV gH or isotype control IgG were added overnight at 4°C to cellular lysates and then protein A/G Sepharose was added for 4 h at 4°C. Protein A/G Sepharose beads with bound protein complexes were spun down, washed with a lysis buffer and resuspended in sample buffer. Western blot analyses were performed using antibodies recognizing β1 and β3 integrins, as well as the HCMV gH and pUL130. Lysates from HCMV-infected monocytes were also analyzed for equal levels of β1, β3 integrins and actin in samples undergoing immunoprecipitation. All experiments were repeated at least three times and representative results are shown. Note: The arrows point to the band of interest. The asterisks mark non-specific bands. (K) The schematic diagram describes our cumulative data from the immunoprecipitation analysis; illustrating the interaction between the gH/gL/UL128-131 complex of TB40/E strain or gH/gL/(gO) complex of AD169 strain with cellular integrins.

Our results demonstrated that the virus functions as a specific ligand that, through the engagement of integrins and activation of downstream signal transduction pathways, is able to modulate monocyte biology. However, the specific ligand on the viral lipid membrane responsible for inducing the integrin/Src-signaling pathway has not been revealed. In an attempt to answer this question, we began to focus on the HCMV gH complexes, as they were shown to be the predominant HCMV glycoprotein complexes reported to interact with integrins on fibroblasts [28], and because the gH/gL/UL128-131 complex was documented to be required for endothelial/epithelial and monocyte tropism [30], [31], [34], [35], [45]. We first investigated if there was a correlation between the presence of the gH/gL/UL128-131 complex on the viral envelope and the ability of several HCMV strains to induce the key integrin/Src-signaling pathway. In our studies, we used HCMV strains containing the gH/gL/UL128-131 complex [our low fibroblast-passaged Towne (Towne/E p.40), a moderately fibroblast-passaged Towne/E (Towne/E p. 51) and TB40/E [39]], as well as viral strains lacking this complex [a highly fibroblast-passaged Towne (Towne/F p. 57), AD169 and TB40/F (a high passage TB40/E [39])]. Using a monoclonal antibody recognizing pUL130 (utilized as a surrogate marker for the UL128-131 complex), we found in virus lysates that the TB40/E strain possessed the highest level of pUL130 and that the continuous propagation of the Towne/E strain in fibroblasts caused a stepwise reduction in the expression of pUL130, such that the protein was no longer detectable in our Towne strain by passage 57 (Towne/F p.57; Figure 1A, lower panel). pUL130 was also not detected in fibroblast-adapted strains (AD169 and TB40/F). These results are in accord with previous reports demonstrating genetic changes in the UL128-131 region in clinical isolates propagated in fibroblasts [36], [39]. As an internal control, we examined levels of the HCMV tegument protein, pp65 (pUL83), in viral lysates (Figure 1A, upper panel). Similar amounts of pp65 were detected in each of the viral strains, suggesting similar particle to PFU ratios were used during infection of cells with the different viral strains. Monitoring of infected fibroblasts with the different virus strains supports our suggestion of a similar number of infectious particles per strain.

Next, we tested our panel of HCMV strains for their ability to induce integrin-mediated signaling. Src tyrosine kinases are established regulators of cellular signaling mediated by integrins [46]–[49], however their activity can also be initiated by other cellular receptors [50]–[53]. Similarly to our previous results [27], here we show that infection of monocytes with Towne/E p.40 caused an increase in the phosphorylation of Src at Tyr416 (∼2 fold increase), when compared to mock-infected monocytes (Figure 1B). Infection with TB40/E had a stronger effect on Src activation (∼3 fold increase over mock); than that observed following infection with Towne/E p.40 (Figure 1B), which correlated with a higher amount of pUL130 in the TB40/E viral particle *vs.* that seen in the Towne/E p.40 viral particle (Figure 1A). The highly passaged HCMV strains lacking a functional gH/gL/UL128-131 complex, however, were unable to initiate Src phosphorylation (TB40/F) or were inhibitory to Src activation (Towne p.57 and AD169) upon infection of monocytes (Figure 1B). In addition, the initial activation of Src by Towne/E p.40 and TB40/E resulted in the activation of a downstream signaling cascade, measured by phosphorylation of p70 S6 kinase (p70 S6K) at Thr389 (4.6- and 6.2-fold increase, respectively, over those levels seen in mock-infected cells; Figure 1B).

Because the activation of the integrin/Src-signaling pathway was demonstrated to be critical for efficient HCMV entry into monocytes [27], we next investigated the ability of HCMV strains expressing different levels of the gH/gL/UL128-131 complex to efficiently enter monocytes. We only observed efficient entry of Towne/E p.40 and TB40/E into monocytes, as indicated by the presence of internalized viral genomic DNA (Figure 1C). In contrast, the signal from the internalized HCMV genomic DNA was significantly lower in cells infected with Towne/F p.57, AD169 and TB40/F (Figure 1C). Equal loading of samples was ensured by examining the level of GAPDH expression (Figure 1C). Together, our results suggest a link between the presence of the functional gH/gL/UL128-131 complex on the viral envelope and the ability of HCMV to induce the integrin/Src-mediated signaling pathway required for efficient viral entry into target monocytes. In addition, because we used viral strains differentiated only by passage length (which in turn relates to cell tropism), our data provides support for the specific role that UL128-131 plays in these specific strains (TB40/E vs. TB40/F and Towne/E vs. Towne/F); at present we are not aware of other mutations that may be present in these different passaged strains. These data also support our previous observation of the critical role for the integrin/Src-signaling in efficient HCMV internalization into monocytes [27].

The aforementioned results suggest that the gH/gL/UL128-131 complex may physically engage cellular integrins on the surface of monocytes to initiate receptor-mediated signaling. Furthermore, we speculated that the gH/gL/UL128-131 complex not only bound integrins, but that it might engage only select integrins differentiating the biological effect caused by the gH/gL complex without the UL128-131 trimer from that of the gH/gL complex with the UL128-131 trimer. Thus, we next immunoprecipitated from HCMV-infected monocytes β1 or β3 integrins, receptors previously found to interact with the HCMV virion on these cells [27], as well as on other cell types [28], [54], [55], and investigated if the UL128-131 complex was capable of engaging these integrins. To pull down sufficient amounts of interacting proteins for their visualization using western blot analysis, we utilized the DTSSP crosslinker to stabilize the interactions. We chose this crosslinker as it allows the dissociation of crosslinked complexes by 5% β-mercaptoethanol and a separation of the individual proteins in those complexes using a standard SDS-PAGE analysis [56]. We found that pull down of β1 and β3 integrins from TB40/E- and AD169-infected monocytes resulted in the finding that the gH protein only interacts with β1 integrins (Figures 1D and 1E). As the lack of gH protein in β3 integrin-immunoprecipitate does not necessary mean the lack of interaction between these proteins, the reverse immunoprecipitation was performed. By immunoprecipitating the gH protein from the lysate of TB40/E-infected cells, we confirmed that gH of TB40/E interacts exclusively with β1 integrin (Figure 1F). However, the immunoprecipitation of the gH protein from the lysate of AD169-infected monocytes suggested that this HCMV glycoprotein can interact with both β1 and β3 integrins (Figure 1G). More importantly, we found that pUL130 present in the gH/gL/UL128-131 complex of TB40/E strain engages both β1 and β3 integrins on the surface of monocytes (Figure 1D). As predicted pUL130 was not detected in immunoprecipitates from monocytes infected with AD169 strain (Figure 1E). As a control for a non-specific binding, we performed immunoprecipitation analyses on lysates from infected monocytes using an IgG isotype matched antibody. We did not detect any signal in our immunoprecipitated samples; however the specific antibodies (to gH and pUL130) recognized viral proteins in the input viral lysate (Figure 1H). Additionally, an immunoprecipitation assay performed on mock-infected cells using an antibody recognizing HCMV gH did not result in a pulldown of the β1 and β3 cellular integrins (Figure 1I). In contrast, antibodies recognizing the β1 and β3 integrins did detect the appropriate integrins in the input lysate (Figure 1I). Western blot analysis also determined that there were equal amounts of cellular proteins in cell lysates used in our co-immunoprecipitation experiments (Figure 1J). The data together suggest that different components of the gH/gL/UL128-131 complex interact on the surface of monocytes with the distinct integrins that we previously showed were important for enhanced motility of and efficient entry into target monocytes [27]. This ability of viruses expressing the UL128-131 complex to bind to the β1 integrin (*via* gH) and to both β1 and β3 integrins (*via* the UL128-131 complex) provides new data as to why HCMV requires both integrins for entry into monocytes [27] (Figure 1K). The results also suggest why only a single β-integrin is likely required for HCMV entry into fibroblasts [28], [55]. Our findings may provide an explanation of why β1 integrins are key regulators of monocyte function as described by Yurochko *et al.* [57]. Based on our new results, we speculate that the interaction of the UL128-131 with β1 and β3 integrins may allow for a unique, synchronous engagement and activation of both β1 and β3 integrins by HCMV, which through the creation of the appropriate type and level of integrin-mediated signaling allows for efficient viral entry and the early functional changes in infected monocytes to occur.

### The HCMV gH/gL/UL128-131 complex is critical for the functional activation of the integrin/Src-mediated signaling pathway and for efficient viral internalization into target monocytes

Long-term passaging of HCMV in fibroblasts alters the virus's genetic composition and those changes are not only limited to the UL128-131 region of the genome [34], [37], [58]–[60]. Consequently, in order to determine if the gH/gL/UL128-131 complex is directly responsible for the initiation of integrin signaling, leading to efficient HCMV internalization into monocytes, we decided to utilize two well-characterized bacterial artificial chromosome (BAC)-based, AD169-derived clones: BAD*wt*, which contains a frame shift mutation in UL131A, and BAD*r*UL131, which possesses a repaired UL131 *locus* and thus has a functional gH/gL/UL128-131 complex [31]. We confirmed that pUL130 was only detected in lysates of BAD*r*UL131 virus, and not in lysates from BAD*wt* (Figure S1A). As a control, we used the AD169 strain that, as showed in Figure 1A, does not express the gH/gL/UL129-131 complex and the TB40/E strain that possesses the pentameric complex on its envelope (Figure S1A).

**Figure 2:**
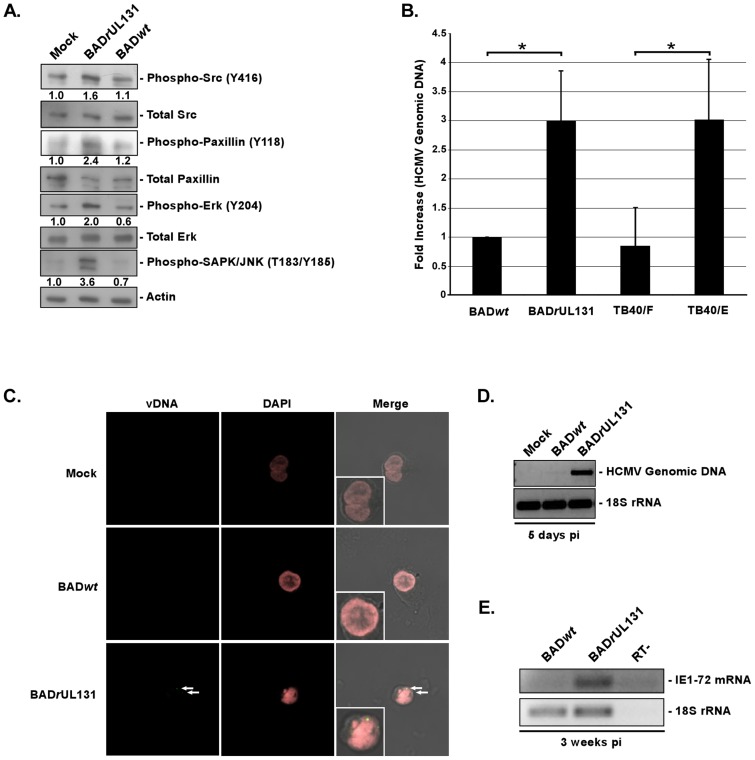
The HCMV gH/gL/UL128-131 complex is critical for activation of, and for efficient and productive viral internalization into target monocytes. (A) Monocytes were cultured in low serum for 24 h at 37°C/5% CO_2_. Monocytes were then mock- or HCMV (BAD*wt* or BAD*r*UL131)-infected (M.O.I. of 5) and harvested at 15 min. pi. Western blot analyses were performed using antibodies specific for the phosphorylated and non-phosphorylated forms of Src, paxillin, Erk and SAPK/JNK. Actin was used as a loading control. The results were also measured by densitometry with relative numbers shown in the figure. (B) Monocytes were HCMV (BADwt, BADrUL131, TB40/F or TB40/E)-infected (M.O.I. of 0.1) for 1 h at 4°C, then temperature shifted to 37°C for 1 h. Monocytes were washed and treated with Proteinase K solution for 1 h. Monocytes were then harvested and quantitative real-time PCR was performed using primers complementary to genomic HCMV DNA and 18S rRNA, as an internal control. Results are plotted as a mean ±SEM. Student's T-tests were performed and p<0.05 (indicated by asterisks) was used for the measurement of statistical significance between samples. (C and D) Monocytes were mock- or HCMV (BAD*wt* or BAD*r*UL131)-infected [M.O.I. of 5 (C) or 1 (D)] and incubated at 37°C in 5% CO_2_ for 5 days. (C) Cells were fixed, cytospun, underwent fluorescence *in situ* hybridization and were analyzed using a confocal microscopy (vDNA – yellow/green & DAPI nuclear stain – pink) or (D) were harvested and semiquantitative PCR was performed using primers complementary to genomic HCMV DNA and 18S rRNA, as an internal control. Note: in the (C) panel, inset pictures are an enlarged version of the representative cell shown in the picture. (E) Monocytes were HCMV (BAD*wt* or BAD*r*UL131)-infected (M.O.I. of 1) and incubated at 37°C in 5% CO_2_ for 3 weeks. RNA was harvested from cells, reverse transcribed and semiquantitative PCR was performed using primers complementary to HCMV IE1-72 mRNA and 18S rRNA, as an internal control. Reverse transcriptase negative (RT-) sample was also tested to demonstrate lack of residual, genomic DNA in samples. All experiments were repeated at least three times and representative results are shown.

We next investigated the ability of these viruses to induce the integrin/Src-signaling pathway. By using western blot analyses, we determined that BAD*r*UL131 was able to increase the level of phosphorylated Src above the levels seen in mock- and BAD*wt*-infected monocytes (Figure 2A). Figure 2A shows a representative western blot experiment with a densitometry analysis, demonstrating 1.6-fold increase of Src activation in BAD*r*Ul131-infected monocytes compared to that seen in mock-infected cells. A cumulative densitometry analysis of Src activation in infected monocytes that incorporates results from three repeats of the experiment showed significant changes in the level of phosphorylated Src in BAD*r*UL131-infected cells, when compared to mock- and BAD*wt*-infected cells (Figure S1B). Furthermore, the initial activation of Src in BAD*r*UL131-infected monocytes translated into the activation of downstream signaling, resulting in increased levels of phosphorylated paxillin (2.4-fold increase), Erk (2-fold increase) and SAPK/JNK (3.6-fold increase), compared to those seen in mock-infected cells (Figure 2A). We also noticed that there was a lower level of phosphorylated forms of Erk and SAPK/JNK in BAD*wt*-infected cells, compared to mock-infected monocytes (Figure 2A), suggesting that BAD*wt* infection may have a slight inhibitory effect on the integrin/Src-signaling pathway in target monocytes. We did not observe any significant differences in levels of total Src, paxillin, or Erk. These results not only substantiate the importance of the gH/gL/UL128-131 complex in the activation of integrin-mediated signaling and validate our previous studies demonstrating the ability of HCMV to engage cellular integrins and to induce the integrin/Src/paxillin signaling pathway in infected monocytes [27], but they further provide insight into how HCMV stimulates integrin receptors on target monocytes.

The data presented so far strongly support a clear correlation between the presence of a functional HCMV gH/gL/UL128-131 complex on the viral envelope and the ability of the virus to trigger integrin/Src-signaling in target monocytes. Therefore, we next examined the ability of BAD*wt* and BAD*r*UL131 to enter target monocytes using HCMV entry assay [26], [27]. Based on the level of internalized vDNA in infected monocytes, we found that BAD*r*UL131 was more efficiently (∼3-fold) internalized into monocytes, when compared to BAD*wt* (Figure 2B). The differences in entry seen between BAD*wt* and BAD*r*UL131 were not due to the ability of these viruses to differentially bind monocytes; we did not observe any significant changes in the binding properties of BAD*wt* and BAD*r*UL131 to monocytes (Figure S1C). We also analyzed the ability of the endotheliotropic TB40/E *vs.* the non-endotheliotropic TB40/F strains to enter monocytes. TB40/F lost its ability to infect endothelial cells, likely due to a frameshift mutation in UL128 gene region [37], [39], [59], as a result of its prolong passaging in fibroblasts. Our results showed that TB40/E was more efficiently (∼3 fold increase) internalized into monocytes than TB40/F. The efficiency of TB40/E internalization into monocytes was similar to that observed following infection with BAD*r*UL131 (Figure 2B). As a control, infected cells were also kept at 4°C without a temperature shift to 37°C. Using this assay, we determined that basal levels of HCMV genomic DNA in cells kept at 4°C was comparable to the levels of BAD*wt* and TB40/F internalization into monocytes at 37°C (Figure S2A). We speculate that even though we saw very low level of viral internalization at 4°C, this entry does not result in a productive infection. Additionally, the proteinase K-treatment might not have removed all non-internalized viral particles from the surface of infected cells, which would also have an effect on the levels of viral genomic DNA in cells maintained at 4°C.

To investigate the ability of BAD*wt vs.* BAD*r*UL131 to establish a productive infection, we performed fluorescence *in situ* hybridization (FISH) analysis on HCMV-infected monocytes to monitor the localization/presence of vDNA at 5 dpi. The signal from the fluorescence probe recognizing HCMV vDNA was only found in monocytes infected with BAD*r*UL131, not in monocytes infected with BAD*wt* (Figure 2C; see the inset pictures for a magnified view of the representative cells). A majority of the cells analyzed showed evidence of vDNA staining at 5 dpi (∼80%). As a control, we verified that BAD*wt* and BAD*r*UL131-infected fibroblasts were both positive for vDNA using this probe (DNS). As FISH only allows for an examination of a small number of cells, we also wanted to examine the infected cell population as a whole, thus we additionally conducted semi-quantitative PCR and RT-PCR analyses looking at HCMV genomic DNA and HCMV IE mRNA expression at 5 dpi and 3 weeks pi, respectively. The results obtained from these experiments support and extend the FISH data; we saw amplification of the HCMV UL123 genomic sequence only from DNA isolated from BAD*r*UL131-infected cells at 5 dpi (Figure 2D). Similarly, HCMV IE mRNA was only found expressed at 3 weeks pi in monocytes/macrophages infected with BAD*r*UL131 (Figure 2E), suggesting that only monocyte-derived macrophages initially infected as monocytes with BAD*r*UL131 were able to express vRNA and, thus were the only cells productively infected. Taken together, our data indicate that the presence of the gH/gL/UL128-131 complex on the HCMV envelope is important for the activation of virus-induced, integrin/Src-mediated signaling pathway in target monocytes and for the efficient viral internalization into these cells that ultimately results in productive viral infection.

### The integrin/Src/paxillin-mediated signaling pathway is critical for BAD*r*UL131 internalization

**Figure 3:**
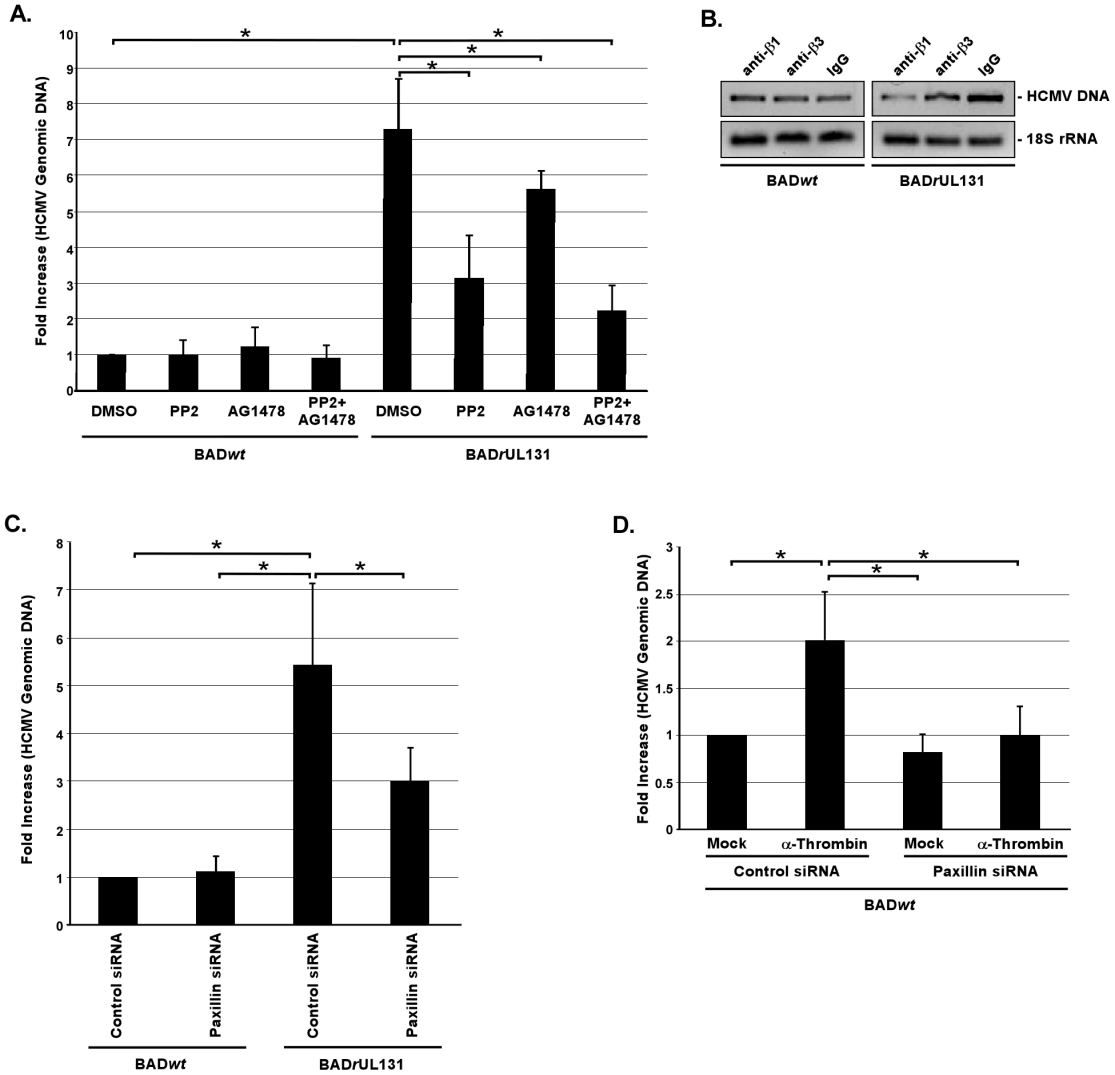
Integrin engagement and activation of the integrin/Src/paxillin signaling pathway by the gH/gL/UL128-131 complex allows efficient HCMV internalization into target monocytes. (A and B) Monocytes were pretreated with 1 µM PP2, 1 µM AG1478, 5 µg/ml of blocking anti-β1 or anti-β3 integrin antibodies, or 5 µg/ml of IgG for 1 h at 37°C/5% CO_2_. (C and D) Monocytes were transfected with siRNA complementary to paxillin or a control siRNA for 48 h. (A, B, C, and D) Monocytes were then HCMV (BADwt or BADrUL131)-infected (M.O.I. of 0.1) for 1 h at 4°C, washed, treated with 5 U/ml of α-thrombin (D only), then temperature shifted to 37°C for 1 h. Monocytes were washed and treated with Proteinase K solution for 1 h. Monocytes were then harvested and quantitative real-time PCR was performed using primers complementary to genomic HCMV DNA and 18S rRNA, as an internal control. Results are plotted as a mean ±SEM. Student's T-tests were performed and p<0.05 (indicated by asterisks) was used for the measurement of statistical significance between samples. All experiments were repeated at least three times.

We have documented that the integrin/Src/paxillin signaling axis must be functionally activated for HCMV to enter blood monocytes [27], and we now demonstrate that the gH/gL/UL128-131 complex is the key trigger for this activation of integrin-mediated signaling and for efficient internalization (Figures 1 and 2). However, because the signaling networks in HCMV-infected monocytes are complex in their nature and involve crosstalk between different receptors [18], [22], [25]–[27], [61]–[63], we wanted to clarify if the Src-mediated signaling pathway interacted molecularly with the EGFR-mediated pathway. Both pathways were shown to be important for efficient HCMV internalization into monocytes; however, both pathways were also found to have a distinct role in regulating the biology of HCMV-infected monocytes [26], [27]. Our new results showed that pretreatment of monocytes with PP2 (specific Src tyrosine kinase inhibitor) and/or AG1478 (specific EGFR tyrosine kinase inhibitor) did not affect the efficiency of BAD*wt* internalization (Figure 3A). However, when monocytes were pretreated with PP2 or AG1478 prior to BAD*r*UL131 infection, viral internalization was inhibited by approximately 56% and 25%, respectively, compared to DMSO-pretreated, BAD*r*UL131-infected cells (Figure 3A). The addition of both PP2 and AG1478 prior to BAD*r*UL131 infection blocked viral internalization by more than 70% compared to DMSO-pretreated, BAD*r*UL131-infected monocytes, however, the cumulative effect of both drugs was not significantly different from the effect of PP2 alone (Figure 3A).

To test for the importance of integrin engagement by the gH/gL/UL128-131 complex in the efficient HCMV entry into monocytes, we pretreated cells with function blocking antibodies to β1 or β3 integrins prior to infection with BAD*wt* and BAD*r*UL131. As shown by the level of internalized vDNA, we determined that this pretreatment did not have any effect on BAD*wt* entry into target monocytes; however the blocking of β1 or β3 integrins inhibited the ability of BAD*r*UL131 to enter these cells by ∼75% or ∼40%, respectively, as determined by densitometry analysis (Figure 3B). The effect of function blocking antibodies on BAD*r*UL131 internalization correlated with their inhibitory impact on the activation of the integrin/Src/paxillin signaling pathway in monocytes infected with HCMV expressing the pentameric complex (Figure S2B and [27]). We did not detect any effect of these blocking antibodies on the integrin/Src/paxillin signaling axis in cells infected with BAD*wt* (Figure S2C). Together, these data provide additional support for the idea that the functional activation of integrin/Src/paxillin signaling pathway, triggered by the HCMV gH/gL/UL128-131 complex, has a causative effect on efficient HCMV entry into target monocytes. It also suggests that even though BADrUL131 predominantly utilizes the integrin-mediated signaling for its internalization into monocytes, the EGFR-mediated signaling also plays at least a supporting role in this process.

We reported earlier that HCMV infection leads to increased expression of paxillin in target monocytes *via* integrin/Src-signaling [27]. We also demonstrated that by knocking down paxillin expression, we were able to significantly decrease HCMV entry into monocytes [27]. To assess the importance of paxillin regulation in BAD*wt vs.* BAD*r*UL131 internalization into monocytes, we used siRNA to knock down paxillin expression as previously described [27] and as before we accomplished a paxillin knockdown efficiency of ∼80–90% (Figure S2D). Using our entry assay, we found that the lack of paxillin expression did not influence BAD*wt* entry into monocytes, while in contrast, BAD*r*UL131 internalization into paxillin-deficient monocytes was inhibited ∼45% (Figure 3C). To attempt to answer if the lack of paxillin activation might be responsible for the inability of BAD*wt* to efficiently enter monocytes, a rescue experiment using α-thrombin, documented to increase phosphorylation of Src and paxillin [64], [65], was also performed. We found that treatment of monocytes with α-thrombin was able to increase the levels of the phosphorylated forms of paxillin and Src with the peak of this activation at 15 min. post the α-thrombin treatment (Figure S2E and data not shown). Because our results demonstrated that the kinetics of HCMV- and α-thrombin-mediated activation were similar (Figure S2E and [27]), we wanted to mimic the pace of paxillin phosphorylation triggered by the gH/gL/UL128-131 complex by exposing BAD*wt*-infected monocytes to α-thrombin just before shifting temperature from 4°C to 37°C in our entry assay. We found that the efficiency of BAD*wt* internalization into monocytes with a normal paxillin expression was significantly (2-fold) enhanced by α-thrombin treatment (Figure 3D). Because α-thrombin can stimulate other signal transduction pathways [66]–[69], we wanted to ensure that α-thrombin-mediated effect on BAD*wt* internalization was paxillin-dependent. Thus, we examined the effect of α-thrombin on BAD*wt* internalization into monocytes deficient of paxillin expression. Monocytes were transduced with scrambled siRNA or siRNA specific for paxillin mRNA, as previously described [27]. The positive effect of α-thrombin on the entry of BAD*wt* into control siRNA-transduced monocytes was diminished in monocytes lacking paxillin expression (Figure 3D), thus suggesting that the inducing effect of α-thrombin on BAD*wt* internalization was paxillin-dependent. In summary, these data support the critical role for the activated integrin/Src/paxillin-signaling pathway, induced through the interaction of integrins with the HCMV gH/gL/UL128-131 complex, in efficient HCMV internalization into monocytes.

### Fibroblasts infected by BAD*wt* and BAD*r*UL131 show an opposite activation pattern when compared to infected monocytes

**Figure 4:**
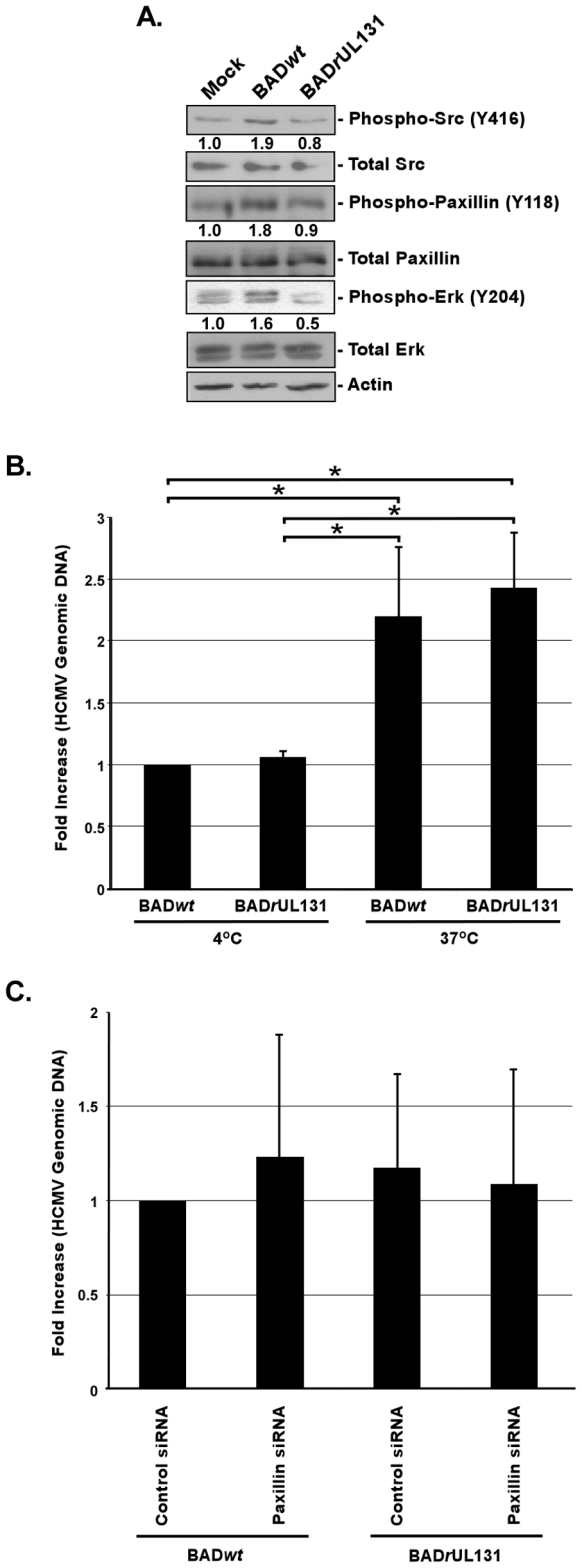
Presence of the gH/gL/UL128-131 complex has no effect on the integrin/Src/paxillin signaling pathway in and HCMV internalization into fibroblasts. (A) Fibroblasts were cultured in low serum for 24 h at 37°C/5% CO_2_. Fibroblasts were then mock- or HCMV (BADwt or BADrUL131)-infected (M.O.I. of 5) and harvested at 20 min. pi. Western blot analyses were performed using antibodies specific for the phosphorylated and non-phosphorylated forms of Src, paxillin, and Erk. Actin was used as a loading control. The results were also measured by densitometry with relative numbers shown in the figure. (B) Fibroblasts were infected with BADwt or BADrUL131 (M.O.I. of 0.1) for 1 h at 4°C, then left at 4°C or temperature shifted to 37°C for 1 h. (C) Fibroblasts were transfected with siRNA complementary to paxillin or a control siRNA for 48 h. Fibroblasts were infected with BADwt or BADrUL131 (M.O.I. of 0.1) for 1 h at 4°C, then temperature shifted to 37°C for 1 h. (B and C) Then, fibroblasts were washed and treated with Proteinase K solution for 1 h. Cells were then harvested and quantitative real-time PCR was performed using primers complementary to genomic HCMV DNA and 18S rRNA, as an internal control. Results are plotted as a mean ±SEM. Student's T-tests were performed and p<0.05 (indicated by asterisks) was used for the measurement of statistical significance between samples. All experiments were repeated at least three times and representative results are shown.

Fibroblasts are a very well studied *in vitro* model system of HCMV infection. Although we have previously shown that HCMV receptor/ligand engagement activates fibroblasts [25], we have also documented that there are key differences between the nature and duration of the signaling during HCMV infection of monocytes *vs.* fibroblasts. For example, HCMV infection of monocytes results in the induction of cellular differentiation, long-term cellular survival, and PI(3)K-independent HCMV entry into monocytes [17], [18], [26], [61], [70], which is not observed in fibroblasts. Additionally, HCMV clinical isolates were reported to infect fibroblasts less efficiently than highly passaged laboratory adapted strains with the opposite being true for the infection of endothelial cells [71], [72], suggesting to us that the presence of the gH/gL/UL128-131 complex differentially affects fibroblasts *vs.* monocytes. Thus, we next investigated if there were differences in receptor-mediated signaling in BAD*wt*- *vs.* BAD*r*UL131-infected fibroblasts. Using western blot analyses, we demonstrated that in fibroblasts, BAD*wt* triggered an increase in the level of phosphorylated Src (1.9-fold increase) as compared to mock-infected fibroblasts (Figure 4A). This initial activation of Src induced downstream signaling, resulting in increased levels of activated paxillin (1.8-fold increase) and Erk (1.6-fold increase) as compared to mock-infected cells (Figure 4A). In contrast, the levels of activated Src and paxillin were not changed in BAD*r*UL131-infected fibroblasts, compared to mock-infected cells (Figure 4A). Interestingly, the level of phosphorylated Erk in fibroblasts infected with BAD*r*UL131 was lower than that seen in mock-infected cells (Figure 4A), suggesting that BAD*r*UL131 may even have an inhibitory influence on molecules downstream of integrin/Src/paxillin-signaling in fibroblasts. As showed by our entry assay, the differences in stimulating the receptor-mediated signaling in fibroblasts by BAD*wt vs.* BAD*r*UL131 did not translate into differences in the ability of these two viruses to be internalized (Figure 4B), supporting previously published data [34].

Because of the importance of paxillin regulation for HCMV entry into monocytes, we assessed the role paxillin plays in BAD*wt vs.* BAD*r*UL131 internalization into fibroblasts. siRNA technology was used to knock down paxillin expression and, similarly to monocytes (Figure S2D and [27]), we were able to decrease paxillin expression in siRNA-transfected fibroblasts by 80 to 90% (Figure S2F). We found that the expression of paxillin did not influence either BAD*wt* or BAD*r*UL131 entry into fibroblasts (Figure 4C), suggesting that although BAD*wt* triggers paxillin phosphorylation during infection of fibroblasts (Figure 4A), paxillin regulation is not required for efficient viral internalization into this cell type. To assure that the differences seen in the ability of BAD*wt* and BAD*r*UL131 to stimulate the integrin/Src-signaling in fibroblasts, as well as the similar abilities of these viruses to enter fibroblasts, was not attributed to differences in binding of BAD*wt vs.* BAD*r*UL131 to fibroblasts, we performed a binding assay. We did not observe significant changes in the binding of the BAD*wt* and BAD*r*UL131 to fibroblasts (Figure S1D).

### Regulation of the actin cytoskeleton is important for efficient entry of HCMV into monocytes

Results presented earlier in this manuscript demonstrated that the functional regulation of paxillin in target monocytes is central for efficient internalization of HCMV virions possessing the gH/gL/UL128-131 complex. We have also recently documented that paxillin expression at the level of mRNA and protein is elevated upon HCMV infection of monocytes, and its regulation is critical for HCMV-mediated pathological motility of target monocytes [27]. Paxillin is a scaffolding and signal transduction protein that plays an important role in regulating the interaction between multiple proteins involved in cell motility and adhesion [73]. Increased paxillin (Tyr118) phosphorylation has been shown to enhance actin polymerization and cytoskeleton rearrangement [74], [75]. Thus, we hypothesized that paxillin is a central regulator of HCMV-mediated changes in infected monocytes and through its role in actin remodeling governs a “hyper” motility of and HCMV entry into monocytes. The role of paxillin-mediated actin rearrangement in cellular motility has been extensively studied [76]–[78], while the importance of paxillin in rapid actin filament rearrangement during viral entry remains unknown. The modulation of the actin cytoskeleton is utilized by several viruses to enhance their infectivity (i.e. Epstein Barr Virus, Vaccinia virus [79]) and entry (i.e. Human Immunodeficiency Virus, Adenovirus [80], [81]), suggesting that HCMV may also require actin rearrangement as a part of its entry process.

Therefore, we asked if HCMV modulates actin rearrangement in infected monocytes. G-actin is a monomeric form of actin, which can polymerize in an ATP-dependent manner to create conformationally changed F-actin-based filaments [82]. Based on western blot analysis, we found that at 15 min. pi, the ratio of F-actin to total actin fell by approximately 50%, suggesting a decrease in polymerized F-actin in HCMV-infected monocytes when compared to mock-infected cells (Figure S3A). Our results also showed that there was a higher level of F-actin at 60 min. pi in HCMV-infected monocytes when compared to mock-infected cells (∼50% higher; Figure S3A), which corresponds to the increased motility of these cells at later times post infection [17], [18], [26], [27], [61]. As a control for our experiments, monocytes were also treated with jasplakinolide (an inducer of actin polymerization) and with latrunculin A (an inhibitor of actin polymerization) [83], [84]. We found that at 15 min. post treatment, jasplakinolide increased the ratio of F-actin to total actin by approximately 40%, and latrunculin A decreased this ratio by 35% when compared to untreated monocytes (Figure S3B).

The presented data suggest that HCMV induces actin rearrangement very early post infection, correlating with the early time frame of viral internalization ([85] and our unpublished results). Because we showed that HCMV was characterized by a lower efficiency of internalization in paxillin-deficient monocytes [27], we next wanted to investigate if the deficiency in paxillin expression affected actin rearrangement. By comparing levels of F-actin to total actin in siRNA-treated monocytes, we demonstrated that cells transfected with paxillin siRNA for 48 h were characterized by a 40–50% decrease in the ratio of F-actin to total actin when compared to this ratio in mock- and control siRNA-treated monocytes (Figure S3C). These results support the idea that paxillin, *via* its regulation of the actin cytoskeleton in monocytes, could be involved in viral entry and that its loss leads to the suppression of normal actin turnover and a decrease in actin polymerization [86]–[89].

**Figure 5:**
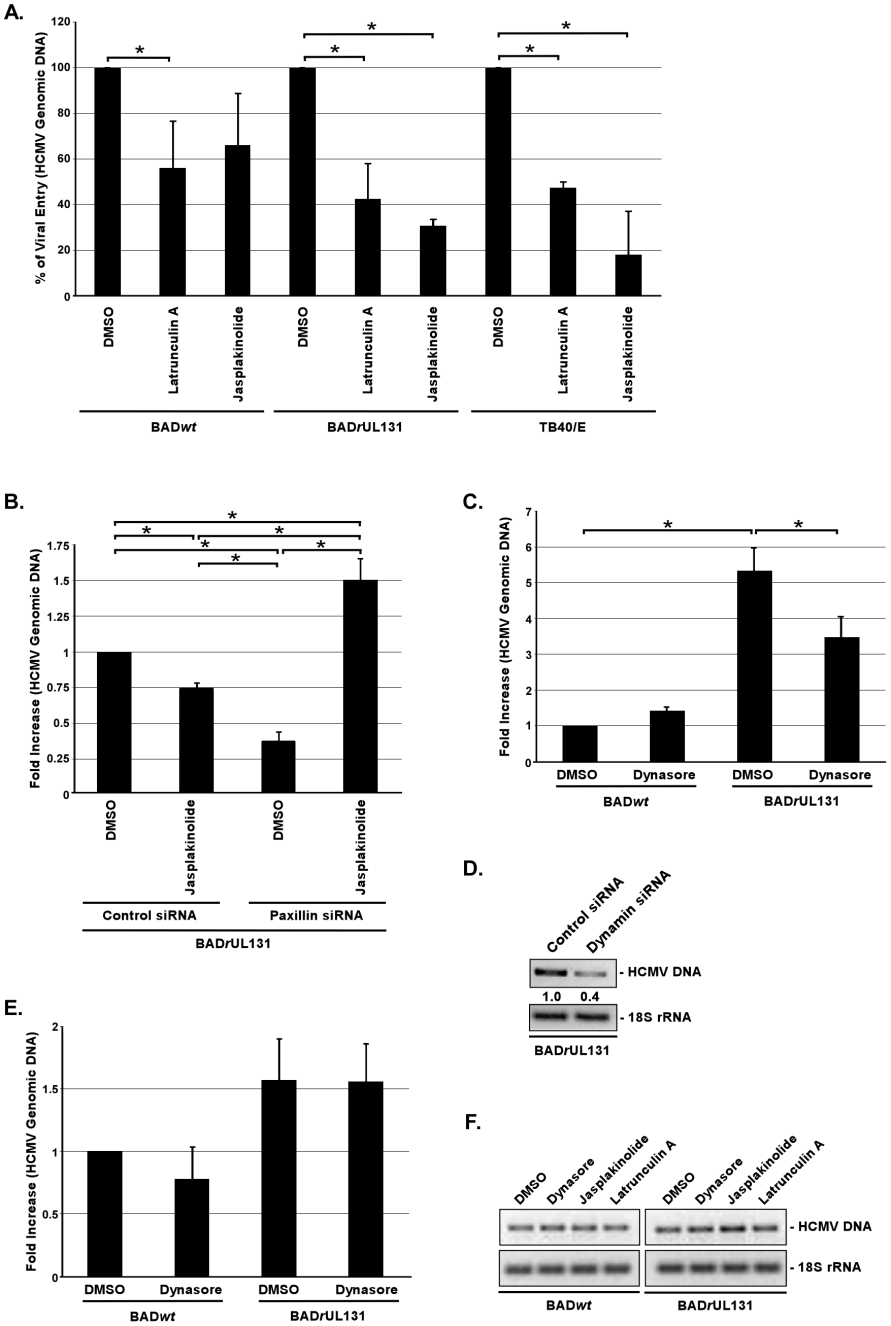
Regulation of the actin cytoskeleton and dynamin is essential for efficient internalization of clinical-like HCMV isolates into monocytes, but not into fibroblasts. (A) Monocytes were pretreated with DMSO, 0.5 µM jasplakinolide, or 2.5 µM latrunculin A. (B) Monocytes were transfected with siRNA complementary to paxillin or a control siRNA for 48 h. (C) Monocytes were preatreated with 50 µM dynasore. (D) Monocytes were transfected with siRNA complementary to dynamin or a control siRNA for 48 h. (E and F) Fibroblasts were pretreated with DMSO, 0.5 µM jasplakinolide, 2.5 µM latrunculin A, or 50 µM dynasore. (A, B, C, D, E, and F) Cells were then infected with BADwt, BADrUL131 or TB40/E (M.O.I. of 0.1) for 1 h at 4°C washed and then temperature shifted to 37°C for 1 h. Monocytes were then treated with 0.5 µM jasplakinolide at 37°C for an additional 1 h (only B). Cells were then washed and treated with Proteinase K solution for 1 h. Cells were harvested and PCR was performed using primers complementary to genomic HCMV DNA and 18S rRNA. For qPCR data, results are plotted as a mean ±SEM. Student's T-tests were performed and p<0.05 (indicated by asterisks) was used for the measurement of statistical significance between samples. For semiquantitative PCR, PCR reactions were analyzed by agarose gel electrophoresis using ethidium bromide. The experiments were performed at least three times.

To examine the direct role of actin rearrangement in the HCMV internalization process into monocytes, cells were pretreated with jasplakinolide or latrunculin A 1 h prior to infection with BAD*wt*, BAD*r*UL131 or TB40/E. From an examination of internalized vDNA, we found that latrunculin A significantly inhibited BAD*r*UL131 (∼60% decrease) and TB40/E (∼53% decrease) entry into monocytes compared to DMSO-treated cells (Figure 5A). When monocytes were pretreated with jasplakinolide, BAD*r*UL131 internalization into monocytes was decreased by ∼70% and TB40/E entry was decreased by more than 80% (Figure 5A). Both pharmacological compounds also had an impact on the internalization process of BAD*wt*; however, the effects were not as substantial as that seen following infection with BAD*r*UL131 and TB40/E. Latrunculin A decreased BAD*wt* internalization efficiency by ∼45%, and jasplakinolide was able to decrease this process by 34%; however, this result was not statistically significant (Figure 5A).

The results presented so far suggest that paxillin, through its regulation of the actin cytoskeleton, affects HCMV internalization into monocytes. To address paxillin's possible direct influence of HCMV entry into monocytes *via* actin rearrangement, we examined if we could rescue the low entry efficiency of BAD*r*UL131 into paxillin-deficient monocytes ([27] and Figure 3C) by inducing actin polymerization in these cells. Shelhass *et al.* [90] has demonstrated that human papillomavirus (HPV) localizes to long, tubular invaginations of plasma membrane in cells treated with cytochalasin D (an inhibitor of actin cytoskeleton dynamics). These structures were shown to hold several viral particles and are thought to emerge due to the inability of endocytic vesicles to pinch off. We hypothesized that HCMV infection of paxillin-deficient monocytes may mimic aspects of HPV entry and thus we could induce the completion of the HCMV entry process into paxillin-deficient monocytes by inducing actin polymerization. Monocytes were treated with siRNA recognizing paxillin mRNA or control siRNA for 48 h and then infected at 4°C with BAD*r*UL131. After an hour-long-incubation at 37°C; cells were treated with an inducer of actin polymerization (jasplakinolide) for an additional hour. Again, we found that BAD*r*UL131 was not able to efficiently enter paxillin-deficient monocytes (Figure 5B). Importantly, jasplakinolide increased (∼4×) the internalization of BAD*r*UL131 in paxillin-deficient monocytes compared to that seen in monocytes treated only with paxillin siRNA (Figure 5B). The level of BAD*r*UL131 internalization in jasplakinolide-treated cells was higher than that seen in control siRNA-treated cells (Figure 5B), which may suggest that there is a concentration of HCMV particles inside plasma membrane structures when paxillin expression is limited, that in turn fully enter when actin polymerization is induced. Noteworthy, we saw an inhibitory effect of jasplakinolide-treatment on BAD*r*UL131 internalization into control siRNA-transfected monocytes (Figure 5B), which may suggest that not all viral particles entered monocytes within the first hour at 37°C. Together, these data strongly support the notion that HCMV utilizes a paxillin-regulated actin rearrangement as a mechanism for its efficient entry into monocytes. It also indicates that there is an interrelationship between the presence of the gH/gL/UL128-131 complex on the HCMV envelope and the dependence of HCMV entry on actin regulation. Moreover, these studies provide new clues about the molecular mechanisms for how HCMV mediates enhanced motility of and efficient viral entry into target monocytes.

### The efficient internalization of HCMV in target monocytes is dynamin-dependent

The involvement of actin rearrangement in the internalization process of HCMV into monocytes indicates that the virus might use one of the endocytic pathways to enter these cells [91]. To address this possibility, we tested a panel of pharmacological inhibitors targeting different modes of endocytosis. Monocytes were pretreated with nystatin (disrupts caveolar structure and function [92]), genistein (disrupts caveolae-mediated endocytosis [93]), Rac1 inhibitor (Rho GTPase inhibitor [94]) and dynasore (dynamin inhibitor [95]) prior to BAD*r*UL131 infection. We found that among these inhibitors, only dynasore significantly (∼35%) blocked HCMV internalization into monocytes (Figure 5C and Figure S3D). Additionally, we analyzed the ability of BAD*r*UL131 to enter dynamin-deficient monocytes. Cells were transduced with siRNA recognizing dynamin mRNA or a control siRNA for 48 h. Even though, we only obtained ∼30% dynamin knockdown in monocytes, as determined by examining the level of dynamin mRNA (Figure S3E), BAD*r*UL131 entry into these monocytes, as showed by levels of internalized vDNA using a semiquantitative PCR analysis, was inhibited by 60% (Figure 5D), which provided an even stronger support for the importance of dynamin regulation in efficient HCMV entry into monocytes. We found that dynasore did not have any effect on BAD*wt* entry into monocytes (Figure 5C), suggesting again that only virus expressing the gH/gL/UL128-131 complex is able to utilize a dynamin-dependent entry mode into monocytes, which in turn leads to productive infection of HCMV in monocyte-derived macrophages (Figure 2C, 2D, and 2E). Additionally, we demonstrated that dynasore, jasplakinolide and latrunculin A were not able to inhibit HCMV entry into fibroblasts, as the efficiency of BAD*wt* and BAD*r*UL131 internalization was not altered by drug-pretreatment in these cells (Figure 5E and 5F). Interestingly, we also observed that the role of integrin/Src/paxillin-signaling, the actin cytoskeleton and dynamin regulation in efficient HCMV entry into monocytes matched their role in HCMV internalization into epithelial cells, as seen by levels of internalized BAD*r*UL131 and TB40/E DNA in epithelial cells pretreated with PP2, AG1478, dynasore, jasplakinolide, and latrunculin A (Figure S3F). Taken together, our data indicate that HCMV, expressing the gH/gL/UL128-131 complex, uses a dynamin- and actin-dependent endocytic-like route to enter into target monocytes and that the internalization process of HCMV is strikingly different to that utilized by the virus in fibroblasts, however it closely resembles a type of HCMV entry utilized in epithelial cells.

## Discussion

HCMV has been demonstrated to be an activating stimulus in fibroblasts and endothelial cells [96], [97] and, as our laboratory has shown, in monocytes [16]–[18], [26], [27], [61], [62]. We previously showed that HCMV infection of monocytes leads to a wide range of biological changes that shape the behavior of target monocytes and set them apart from model systems, highlighting HCMV's unique influence on the biology of infected monocytes following primary infection. The biological changes seen in HCMV-infected monocytes allow the virus to use the natural sentinel role of circulating monocytes to exit the blood stream and translocate to multiple host organ tissues, where monocytes, which are non-permissive for viral replication, undergo a distinct HCMV-driven differentiation into macrophages that support viral replication and production of progeny virions [16]–[18], [63], [70]. Our previous data strongly suggested that HCMV binding to target cells triggers specific biological changes *via* receptor/ligand-initiated processes [16]–[18], [25]. HCMV has been shown to bind to several different cellular receptors, with utilization of these receptors by the virus being apparently cell type specific [22], [26]–[29], [45], [55], [98], [99]. We have recently reported that EGFR and integrins were engaged by HCMV on the surface of monocytes resulting in the receptor-mediated signaling pathways found to be critical for efficient HCMV internalization into monocytes, virus-induced “hyper” motility of and the prolonged survival of these cells [22], [26], [27]. Our data indicated that HCMV overcomes its restricted replication in monocytes by inducing EGFR- and integrin-mediated signaling, allowing the virus to use monocytes as virus-carriers for its systemic spread. However, the specific viral trigger responsible for monocyte activation has not been identified.

We proposed that the ability of HCMV to induce distinct signal transduction pathways resulting in functional changes in monocytes is determined by the nature of the viral glycoproteins expressed on the mature viral envelope and, specifically, by the presence of the HCMV gH/gL/UL128-131 pentameric complex. The complex is required for endothelial/epithelial cell infection, virus transfer to leukocytes and infection of monocytes [30], [31], [34], [35], [45]. Nevertheless, the mechanism allowing the gH/gL/UL128-131 complex to promote the viral entry process has not been revealed. It has been proposed that the UL128-131 complex dictates tropism simply due to the ability of gH/gL/UL128-131 complex to bind to different cell types [41]. We postulate that this region not only dictates binding, but it also governs the type and/or levels of receptor-mediated signaling.

To investigate the role of the gH/gL/UL128-131 pentamer in the ability of HCMV to induce signal transduction pathways in target monocytes, we first used several HCMV strains that differed in the presence of the gH/gL/UL128-131 complex. We found that only viruses expressing the gH/gL/UL128-131 complex were able to activate integrin/Src-signaling and be efficiently internalized into target monocytes. Therefore, our data established a functional connection between the presence of the gH/gL/UL128-131 complex on the mature HCMV envelope, the receptor-mediated signal transduction and the ability of HCMV to be efficiently internalized into target monocytes. These results supported our previous report documenting the critical role of integrin/Src-signaling for efficient HCMV internalization into monocytes [27]. The current report argues for the first time that the gH/gL/UL128-131 complex plays a role as a specific ligand for activating monocytes and, consequently, allowing for viral entry into these cells.

While looking at the molecular mechanism of the interaction between the gH/gL/UL128-131 complex and cellular integrins, we determined that the gH/gL/UL128-131 complex enables HCMV to simultaneously interact with two distinct heterodimeric integrins on the surface of monocytes. Specifically, gH only interacts with β1 integrins, which have been demonstrated to be central regulators of cellular immediate-early gene induction in monocytes [57], and the UL128-131 trimer engages both β1 and β3 integrins. gH from the gH/gL/(gO) complex was able to engage both β1 and β3 integrins on monocytes, however this engagement did not result in the induction of integrin-mediated signaling in infected monocytes. We speculate that the gH/gL/UL128-131 complex may provide a more effective interaction with cell surface receptors and therefore create a higher affinity binding, which in turn induces a higher level of cellular activation. Additionally, the UL128-131 complex may also interact with different regions of the cellular integrins, allowing for augmented integrin-mediated signaling seen in infected monocytes. We argue that HCMV uses the pentameric gH complex to execute this intrinsic β1 integrin-mediated stimulation of monocytes for its own advantage, while the UL128-131 complex alone allows for the additional required stimulus for integrin-mediated signaling in monocytes. Therefore, the whole gH/gL/UL128-131 complex allows for a synchronous engagement of β1 and β3 integrins by HCMV and the creation of an appropriate type and level of integrin-mediated signaling in target monocytes. The aforementioned results hint that a close spatial cooperation between different integrins and possibly other surface receptors (i.e. EGFR [26]) on monocytes is critical for specific HCMV-mediated changes in monocytes. Wang *et al.* demonstrated that lipid rafts were important for the HCMV-mediated interaction between integrins and EGFR and for orchestrating the receptor-initiated signalosome [28]. Our data also suggest that lipid rafts are important for receptor-mediated signaling in HCMV-infected monocytes (unpublished data).

In our studies, we used two well-characterized viruses expressed from a BAC system: BAD*wt* (an AD169 clone), containing a frameshift mutation in UL131A and thus is without a functional pentameric complex, and BAD*r*UL131, that possesses a repaired and functional gH/gL/UL128-131 complex [31], to better understand the role of the pentameric complex in viral-mediated signaling in and entry into target monocytes. In monocytes, we found that only BAD*r*UL131 was able to induce the integrin/Src activation and their downstream signaling partners, strongly suggesting that the gH/gL/UL128-131 complex is a specific ligand responsible for activating the integrin-signaling pathway. Moreover, we found that the ability of BAD*r*UL131 to initiate monocyte activation translates into a more efficient process of viral internalization into these cells compared to that seen for BAD*wt*. Like BAD*r*UL131, we also found that TB40/E strain was more efficiently internalized into monocytes, as compared to TB40/F. We are aware, however, that Ryckman *et al.* [71] reported a difference in the ability of HCMV lacking the UL128-to-UL150 genes (HCMV TRΔ4 strain) to be absorbed on the surface of epithelial cells and fibroblasts compared to wild type TR strain. These authors did not find a decreased capability of AD169 to bind these cells [71], suggesting that possible proteins associated with the changed absorption rate of HCMV on monocytes, fibroblasts and epithelial cells are not encoded from the UL128-131 gene cluster. Importantly, by monitoring the cellular localization and expression of vDNA and vRNA, our studies also determined that only BAD*r*UL131, but not BAD*wt*, productively infects monocytes. Taken together, our data strongly support the thesis that the presence of the gH/gL/UL128-131 complex on the HCMV envelope is critical for the activation of the virus-induced, integrin/Src-mediated signaling pathway in target monocytes and for efficient viral entry into these cells, as well as for productive infection. Additionally, our report supports and provides a mechanistic explanation to results presented in a recent report showing that the UL128 component of the gH/gL/UL128-131 complex was important in HCMV infectivity of monocytes [35].

To determine the importance of receptor-mediated signaling mediated by the gH/gL/UL128-131 complex in the increased internalization of BAD*r*UL131 compared to BAD*wt*, we tested the role the integrin/Src- and EGFR–mediated signaling pathways played in virus entry. We did not observe any influence of these signaling pathways on BAD*wt* entry into monocytes. In contrast, BAD*r*UL131 internalization was significantly inhibited in monocytes that had both the integrin and/or EGFR signal transduction pathways impeded. Additionally, our studies demonstrated that, unlike for BAD*wt*, β1 and β3 integrins are engaged by BAD*r*UL131 to stimulate the integrin/Src/paxillin-signaling and are critical for efficient BAD*r*UL131 entry into monocytes, supporting our previous report [27]. Our experiments also demonstrated that paxillin regulation plays an important role in BAD*r*UL131 internalization into monocytes, as only the internalization of BAD*r*UL131, and not BAD*wt*, was hindered in paxillin-deficient monocytes. Interestingly, we were able to artificially increase the entry efficiency of BAD*wt*, characterized by a low infectivity rate in monocytes, by inducing α-thrombin-mediated paxillin phosphorylation in these cells. This α-thrombin-mediated effect was abrogated in monocytes lacking paxillin expression, indicating that regulation based on the integrin/Src/paxillin signaling axis is vital for efficient HCMV entry into monocytes. Taken together, these results strengthen our previously stated hypothesis of the important role the viral pentameric complex plays in the induction of the receptor-mediated signalosome required for efficient HCMV entry into target monocytes.

Long-term passaging of HCMV clinical isolates introduces changes into the viral genome [30], [39], [100]–[104]. However, it has not been evident why there is a strong selection against the UL128-131. We believe that our present studies shed light on this matter, as the effect of BAD*wt* and BAD*r*UL131 on the activation of receptor-mediated signaling pathways in fibroblasts was opposite to that seen in infected monocytes. In fibroblasts, it was BAD*wt*, not BAD*r*UL131, which triggered Src phosphorylation and activation of the downstream signaling cascade. Interestingly, we noticed an inhibitory effect of BAD*r*UL131 on Erk activation, which previously was found to be required for efficient HCMV infection [105]–[107]. Additionally, our data indicate that paxillin regulation does not play an important role in HCMV entry into fibroblasts and the different potential of BAD*wt vs.* BAD*r*UL131 to induce the integrin/Src/paxillin-signaling axis in these cells did not influence their ability for internalization, suggesting that the expression of the UL128-131 complex does not provide any advantage to the virus. Thus, this *locus* might be more preferentially mutated when HCMV clinical isolates are passaged in fibroblasts. We noticed that HCMV induces relatively low activation of signaling molecules in both monocytes and fibroblasts (∼2× higher than in mock-infected cells), when analyzed by western blot analysis. We argue that this effect is significant, as we estimate, based on the biology of the virus-cell interactions and our experimental design, that only between 10–20% of cell surface integrins are engaged by HCMV [108], which in turn translates to only ∼10–20% of the total number of Src molecules being activated in an infected cell. Additionally, we observed that higher number of viral particles used for infecting monocytes translated into greater activation of the integrin-mediated signaling in these cells. Taken together, our and others' results suggest that, even though HCMV is capable of activating integrin/Src-signaling in both fibroblasts and monocytes, there are significant differences in the utilization of receptor-mediated signaling networks between these two cell types [26]–[28], [99]. Biologically, these differences likely correspond to the nature of the cell and the role the cell plays during the infection process.

There is a growing list of evidence demonstrating that herpesviruses can utilize different mechanisms of internalization into different cell types. Depending on the cell type, herpes simplex virus (HSV) was shown to enter cells using both pH-dependent and pH-independent fusion with endosomes, as well as direct fusion with the plasma membrane [109]–[111]. Similarly, Miller and Hutt-Fletcher demonstrated that Epstein-Barr virus (EBV) penetrates normal B cells by pH-independent endosomal fusion; however, direct fusion with the plasma membrane is utilized in EBV infection of epithelial cells and transformed B cells [112]. Direct fusion with the plasma membrane was described as a mode of HCMV entry into fibroblasts [113], and endocytosis was postulated to be involved in the HCMV internalization process into epithelial and endothelial cells [41], [85]. Actin has been implicated as a key player in the process of endocytosis [42]–[44]. We have recently documented that the expression of paxillin, a protein regulating actin cytoskeleton dynamics and governing endocytosis [114], is elevated upon HCMV infection of monocytes and is critical for efficient HCMV entry into this cell type [27]. The findings in our current report indicate that this regulatory protein might be used by HCMV in target monocytes as a convergence point that links integrin/Src-signaling to the regulation of the actin cytoskeleton that is needed in the HCMV entry process. The role of paxillin-mediated actin rearrangement in cellular motility has been studied extensively [76]–[78], while the importance of paxillin in rapid actin filament rearrangement during viral entry has remained unknown. Here, we documented that HCMV caused a rapid and transient actin depolymerization in infected monocytes. Moreover, we found that there was a decreased level of polymerized actin in paxillin-deficient monocytes compared to mock- or control siRNA-treated cells, which supports earlier reports showing that increased paxillin phosphorylation enhanced actin polymerization and cytoskeleton rearrangement [74], [75]. Thus, we suggest the importance of actin regulation in HCMV internalization and implicate endocytosis as a possible route of entry into monocytes. We demonstrated that the disruption of the actin cytoskeleton prevents the efficient entry of BAD*r*UL131, TB40/E, indicating that there is an interrelationship between the presence of the gH/gL/UL128-131 complex on the HCMV envelope and the dependence of HCMV entry into monocytes on actin regulation. A disruption of actin regulation did not have any effect on BAD*wt* and BAD*r*UL131 entry into fibroblasts. Thus, our data again provide evidence that the effect of the UL128-131 complex on HCMV entry is cell type specific. Interestingly, we found that we could rescue the inability of BAD*r*UL131 to enter paxillin-deficient monocytes by inducing actin polymerization after viral binding to the cell, suggesting that, similarly to studies on HPV entry [90], by inhibiting actin polymerization in monocytes HCMV may localize to tubular invaginations of plasma membrane due to the inability of endocytic vesicles to pinch off. However, this theory needs further investigation. Together, our data support the notion that a tight regulation of the actin cytoskeleton plays an essential role in the ability of HCMV to efficiently enter monocytes.

The exploitation of actin rearrangement by HCMV points out the possible role of endocytosis in the viral entry process [91]. Using several pharmacological inhibitors targeting different modes of endocytosis, we found dynasore, a dynamin inhibitor, to be a potent agent impeding this process. Moreover, a 60% inhibition of HCMV entry was observed in infected monocytes that were deficient in dynamin expression, which provides an even stronger support for an importance of dynamin regulation in efficient HCMV entry into monocytes. As dynasore lacked an inhibitory effect on HCMV entry into fibroblasts, and with no apparent effect of this drug on BAD*wt* entry into monocytes, we argue that only virus expressing the gH/gL/UL128-131 complex is able to utilize the endocytic entry into monocytes. Consequently, our data indicate that HCMV, expressing the gH/gL/UL128-131 complex, uses a dynamin- and actin-dependent endocytic route of entry into target monocytes, which as our current studies showed is similar to the characteristics of HCMV entry seen in epithelial cells. A similar strategy of viral entry is employed by human adenovirus 2, in which the penton complex interacts with integrins promoting dynamin- and actin-based viral endocytosis [81], [115]–[118]. Thus, HCMV joins the growing list of viruses (i.e. EBV [79], Vaccinia virus [79], HIV [80], Adenovirus [81]) that utilize the actin cytoskeleton to enhance their infectivity.

It has been suggested that, within a number of endocytic pathways, macropinocytosis is the most likely route of HCMV entry into *in vivo* relevant cell types [119]. As HCMV measures 200–300 nm in diameter [120], small vesicles created in clathrin-, caveolea- or micropinocytosis-based uptakes could not accommodate virions of such size; this possibility is also supported by our results. Additionally, EBV and HSV in certain cell types have also been considered to utilize macropinocytosis [110], [111], [121]. Mercer and Helenius compiled the experimental criteria allowing for a characterization of macropinocytosis as a mode of viral entry [91]. Our findings showed that HCMV entry into monocytes is actin rearrangement- and dynamin-dependent, supporting macropinocytosis as the mechanism of entry (the current report). However, we also know that the HCMV entry process into monocytes is not regulated by PI(3)K [26], an important regulator of macropinocytosis [91], suggesting that HCMV may use a non-classical macropinocytosis route for its internalization into target monocytes. Thus, these data again underscore the specificity of the biological processes in HCMV-infected monocytes regulated *via* distinct signaling events.

**Figure 6:**
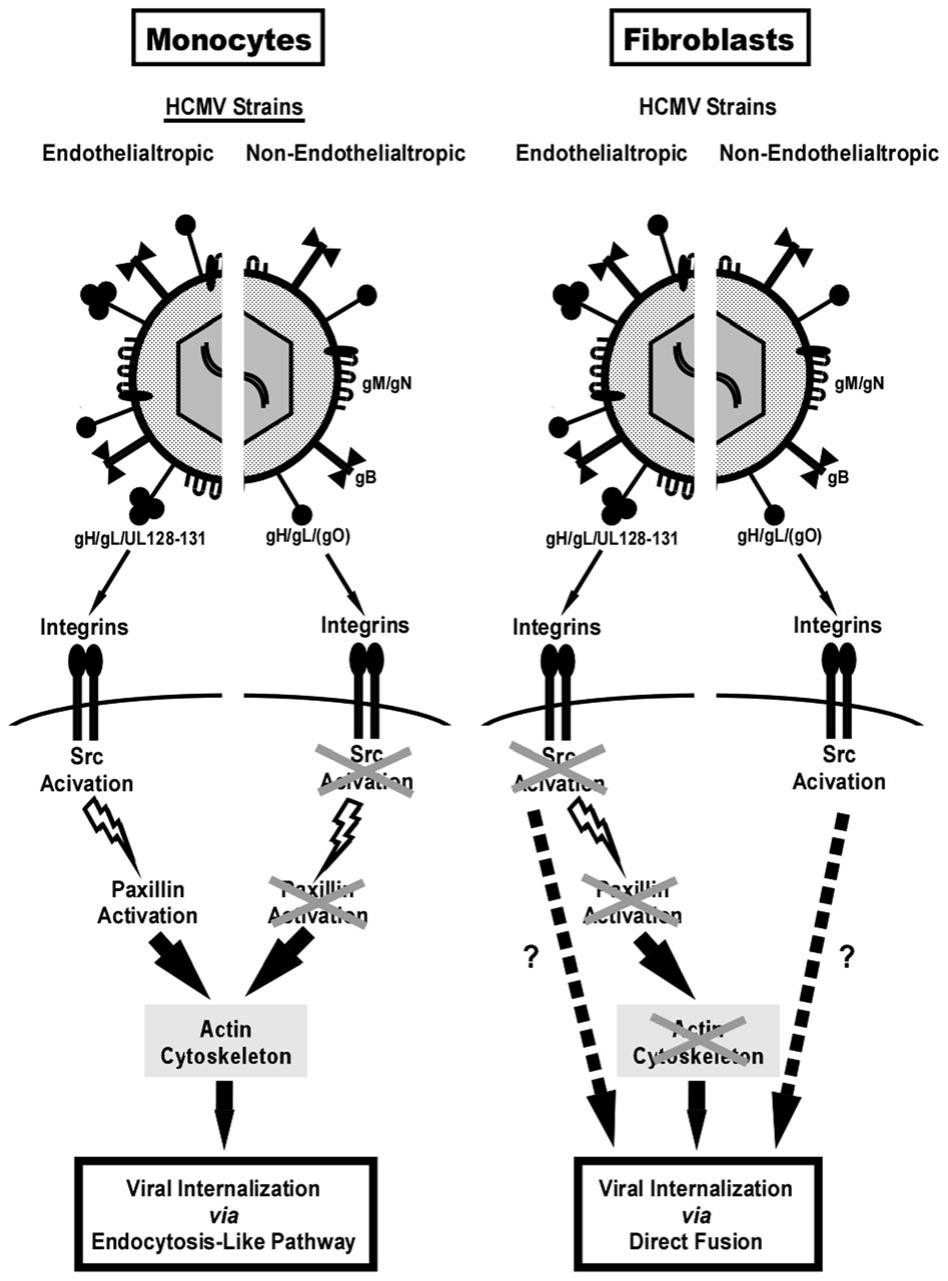
Our model: The HCMV gH/gL/UL128-131 complex differs in its ability to trigger functional cellular activation in monocytes vs. fibroblasts, causing contrasting modes of entry into these cell types. On monocytes, the gH/gL/UL128-131 and gH/gL/(gO) complexes engage both β1 and β3 integrins, however, only the pentameric complex triggers integrin-mediated signaling. In the case of the gH/gL/UL128-131 complex, the gH protein interacts with β1 integrins, and the UL128-131 complex engages both β1 and β3 integrins, suggesting that the UL128-131 complex allows for cooperative regulation of receptor-mediated signaling pathway(s) initiated through the activation of separate integrin heterodimers to create the appropriate type/level of receptor-mediated signaling beneficial to the virus. This signal transduction process is consequently responsible for the functional activation/phosphorylation of paxillin (lightning bolts), which governs the rearrangement of the actin cytoskeleton ultimately leading to efficient internalization of HCMV into monocytes via an endocytosis-like pathway (black arrows). On fibroblasts, the gH/gL/(gO) complex(es) triggers integrin/Src-mediated signaling, however, paxillin-dependent actin rearrangement is not important for HCMV internalization, suggesting an involvement of contrasting molecular pathways in HCMV-infected fibroblasts (dotted arrows) *vs.* those seen in monocytes, leading to different modes of HCMV entry into fibroblasts *vs.* monocytes.

As Figure 6 depicts, our overall data showed that HCMV equipped with the pentameric gH/gL/UL128-131 complex is able to engage integrins on the surface of monocytes and, through the induction of the integrin/Src/paxillin signaling pathway, to regulate actin cytoskeleton, which consequently leads to efficient viral internalization and productive infection in target monocytes. These immediate early biological changes in target monocytes resulting in the appropriately executed entry process further enable HCMV, through prolonged receptor-mediated regulation and virus-enhanced cellular motility [27], to utilize the natural sentinel role of these cells. This mode of action allows HCMV to avoid the host immune response and effectively spread within the host. Scivano *et al.* recently demonstrated that HCMV-infected fibroblasts released a mixture of endothelial cell-tropic (high level of the gH/gL/UL128-131 complex) and non endothelial cell-tropic (low level of the gH/gL/UL128-131 complex) virions and the spread of this virus was supernatant-driven [122]. In contrast, endothelial cells released virions with low levels of the pentameric complex into the supernatant, and virions with a high level of the gH/gL/UL128-131 complex were cell-associated. Therefore, the spread of the endothelial cell-originated virus was predominantly focal. Additionally, we previously showed that HCMV promotes expression of adhesion molecules in endothelial cells, promoting the recruitment of naïve monocytes and augments monocyte transendothelial migration by increasing the permeability of endothelium [123]. Moreover, we found that the virus was transferred to monocytes while translocating through the endothelium [123]. Taken together, the expression of the gH/gL/UL128-131 complex on the viral envelope and the requirement for the appropriate level/type of integrin/Src/paxillin signaling, induced by the pentameric complex in infected cells, might be the evolutionary mechanism that assures the predominance of close proximity HCMV infection minimizing the recognition of virus by the immune system and at the same time allowing for efficient viral spread within the host.

In summary, our findings provide, for the first time, an explanation of why the gH/gL/UL128-131 complex is critical for viral internalization into a clinically relevant cell type, and connects it with the importance of receptor-mediated cellular changes for a successful HCMV infection. Our report also indicates that the inability of the gH/gL/UL128-131 complex to stimulate the integrin/Src/paxillin-mediated signaling pathway in fibroblasts may explain the conundrum of why there is a strong negative pressure against the UL128-131 region during the passaging of clinical isolates in fibroblasts. Therefore, to our knowledge, our studies are the first to demonstrate a possible molecular mechanism for why the gH/gL/UL128-131 complex dictates HCMV tropism.

## Materials and Methods

### Ethics statement

We explicitly state that the Louisiana State University Health Sciences Center-Shreveport (LSUHSC-S) Institutional Review Board (IRB) approved our study (the approved study number is H99-064) and that all HIPAA and LSUHSC-S IRB guidelines were followed for the use of human subjects in our study. In addition, the National Institutes of Health has approved our study using human subjects as part of our funded application (AI050677). We also explicitly state that informed written consent was provided by study participants (using an approved consent form, from the approved protocol H99-064); that is, an approved consent form was signed by the participants and collected for all subjects utilized in our study. No vulnerable populations were utilized in our study (minors, pregnant women, prisoners, etc.); thus only the participants themselves signed the consent approved forms and no legal guardians were required to provide consent to participate in the study. As per HIPAA and LSUHSC-S IRB guidelines, we follow all protocols on the welfare of human subjects and on the privacy of all collected information. The LSUHSC-S IRB is under the guidance of the Human Research Protection Program (HRPP) and is headed by the chancellor of the University, Dr. Robert Barish, M.D. (the website for our HRPP is as follows [http://www.lsuhscshreveport.edu/HRPP/HRPPHome.aspx]). The board is as follows: Institutional Official (Robert A. Barish, MD, MBA); HRPP (Robert E. Walter, MD, MPH; Aliese Seawright, MS, C.I.P; Kathleen Bloomingdale, BS; and Janice Soderstrom, RN, BSN); QA/QI (Sherry Mosura, RN, BSN, CCRC; Yvette Viverette, RN, CCRC); and, IRB (Christina Copeland, MPH; Shweta Khedlekar, MS; Ann Johnson; Crystal McGee).

### HCMV culture and infection

AD169-derived bacterial artificial chromosome (BAC) viruses BAD*wt* and BAD *r*UL131 (containing a repaired UL131A mutation, such that the gH/gL/UL128-131 complex is identical to that of HCMV TR strain), were previously constructed and characterized [31]. In addition to these BAC-based viruses, several additional HCMV strains were used: a green fluorescent protein (GFP)-labeled TB40/E-UL32 (TB40/E) [39]; a high fibroblast-passaged TB40 (TB40/F) that has lost the ability to infect endothelial cells [39]; our Towne/E (with the UL128-131 complex intact; Towne p.40); a intermediate fibroblast-passaged Towne (Towne p.51); and, a high fibroblast-passaged Towne (without a functional UL128-131 complex; Towne p.57) [123]. The TB40/E and TB40/F strains [39] are related (TB40/F is a high passaged TB40/E) as is our Towne/E and Towne/F strains [39]. TB40/E and Towne/E retain endothelial and epithelial cell, as well as monocyte tropism, while TB40/F and Towne/F have lost the ability to infect these clinically relevant cells types and are restricted to fibroblast infection. As shown by the data in Figure 1, those viruses retaining endothelial and epithelial cell, and monocyte tropism possess an intact UL128—131 complex, while those restricted to fibroblast infection have lost this complex. It is currently unclear if additional mutations have been incorporated into the more highly passaged viruses. The viruses were cultured in human embryonic lung (HEL) fibroblasts with 4% heat-inactivated fetal bovine serum (FBS) and purified on a sucrose gradient and resuspended in RPMI 1640 (Cellgro, Mediatech Inc., Herndon, VA). Monocytes were infected with purified virus at the multiplicity of infection (M.O.I.) of 0.1 for entry assays or M.O.I. of 5 for the various functional assays, unless otherwise stated. Mock infection was carried out by adding an equivalent volume of RPMI 1640 (Cellgro) to monocytes.

### Human peripheral blood monocyte isolation

Double-density gradient centrifugation was used to purify human peripheral blood monocytes [57]. Whole blood was collected from donors by venipuncture. Mononuclear cells were then collected by centrifugation through a Ficoll Histopaque 1077 (Sigma-Aldrich, Inc., St. Louis, MO) gradient. Next, the collected cells were washed in 1× Phospho-Buffered Saline (PBS; Cellgro) to remove platelets. Monocytes were then isolated by centrifugation through a Percoll (Pharmacia Biotech, Inc., Piscataway, NJ) gradient and suspended in RPMI 1640 (Cellgro) supplemented with 1% human serum (Sigma-Aldrich Inc.).

### Cell treatment and culture

After isolation, monocytes were cultured under non-adherent conditions (unless specified otherwise) in RPMI 1640 (Cellgro) supplemented with 1% human serum (Sigma-Aldrich Inc.) at 37°C with 5% CO_2_ overnight, prior to any treatment, if not stated otherwise. Primary human embryonic lung (HEL) fibroblasts (passage 13–20) were cultured in Eagle's minimal essential media (MEM, Cellgro) supplemented with 10% heat-inactivated fetal bovine serum (FBS, Gemini, Woodland, CA), penicillin (100 IU/ml; Cellgro), and streptomycin (100 µg/ml; Cellgro) and Plasmocin (25 µg/ml; InvivoGen, San Diego, CA). Primary human mammary epithelial cells (HMEC) were culture in EBM-2 Basal Medium (Cambrex Inc., East Rutherford, NJ). The following standard treatment groups were employed in our study: dimethyl sulfoxide (DMSO; Sigma-Aldrich Inc.) as a solvent control; 1 µM PP2 (Src tyrosine kinase inhibitor; EMB Biosciences, Inc.; La Jolla, CA); 1 µM AG1478 (EGFR tyrosine kinase inhibitor; EMB Biosciences, Inc.); 0.5 µM jasplakinolide (inducer of actin polymerization; Enzo Life Sciences International, Inc.; Plymouth Meeting, PA); 2.5 µM latrunculin A (inhibitor of actin polymerization; Enzo Life Sciences International, Inc.); 50 µg/ml nystatin (disrupts caveolar structure and function; Enzo Life Sciences International, Inc.); 200 µM genistein (disrupts caveolae-mediated endocytosis; Enzo Life Sciences International, Inc.), 100 µM Rac1 inhibitor (Rho GTPase inhibitor; Enzo Life Sciences International, Inc.) and 50 µM dynasore (dynamin inhibitor; Enzo Life Sciences International, Inc.) were added 1 h prior to HCMV infection at 37°C with 5% CO_2_. In one of the viral entry assays, 0.5 µM jasplakinolide was added after the shift in temperature to 37°C. In one of the viral entry assays, 5 U/ml of α-thrombin (Enzyme Research Laboratories, Inc., South Bend, IN) was added prior to the shift in temperature to 37°C. Cells were also treated with 5 µg/ml of function-blocking anti-β1 integrin (Millipore, Bedford, MA) and/or anti-β3 integrin (Millipore) antibodies for 1 h at 4°C prior to HCMV infection, as well as with an IgG isotype control (Santa Cruz Biotechnology, Inc.)

### Western blot analysis

Monocytes or fibroblasts were pretreated with pharmacological agents and then infected. To examine the kinetics of receptor-mediated signaling pathways, cells were harvested using lysis buffer [50 mM Tris-HCl at pH 7.5 (Fisher Scientific, Fair Lawn, NJ), 5 mM ethylenediaminetetraacetic acid (EDTA; BioRad Laboratories, Hercules, CA), 100 mM sodium chloride (Fisher Scientific), 1% Triton X-100 (Fisher Scientific), 0.1% sodium dodecyl sulfate (SDS; MP Biomedicals, Inc., Solon, OH), and 10% glycerol (MP Biomedicals, Inc.)] at the time points indicated. Samples were mixed with Laemmli's SDS-Sample Buffer (Boston BioProducts, Boston, MA) containing 2-mercaptoethanol (Fisher Scientific). Equal protein amounts of the different samples were separated by continuous polyacrylamide gel electrophoresis (SDS-PAGE) and transferred to ImmunoBlot polyvinylidene difluoride (PVDF) membranes (BioRad Laboratories). Western blot analyses were performed using primary antibodies recognizing the phosphorylated and non-phosphorylated forms of Src [phospho-Src (Tyr416) antibody (Cell Signaling Technology, Inc., Danvers, MA) and pan-Src (Santa Cruz Biotechnology, Inc., Santa Cruz, CA)], of paxillin [phospho-paxillin (Tyr18) antibody and pan-paxillin antibody (Cell Signaling Technology, Inc.)], of SAPK/JNK [phospho-SAPK/JNK (Thr183/Tyr185) antibody and pan-SAPK/JNK antibody (Cell Signaling Technology, Inc.)], of Erk [phospho-Erk (204) antibody and pan-Erk1 antibody (Santa Cruz Biotechnology, Inc.)], of p70 S6K [phospho-p70 S6K (Thr389) and pan-p70 S6K antibody (Cell Signaling Technology, Inc.)], as well as antibodies recognizing cellular F-actin (Thermo Fisher Scientific; Rockford, IL), and recognizing HCMV proteins: pUL130 (gift from Dr. Thomas Shenk) and pp65 (Virusys Corporation; Taneytown, MD). Probing for actin (Santa Cruz Biotechnology, Inc.) was used as a loading control. Donkey anti-rabbit (GE Healthcare Life Sciences, Piscataway, NJ), donkey anti-mouse (GE Healthcare Biosciences) and donkey anti-goat (Santa Cruz Biotechnology, Inc.) conjugated with horseradish peroxidase (HRP) were used as secondary antibodies. Western blots were developed using ECL Plus Western Blotting Detection Reagents (GE Healthcare Life Sciences).

### Immunoprecipitation

Monocytes were infected with HCMV at 4°C for 1 h. Subsequently, 2 mM of DTSSP [3,3′-dithiobis(sulfosuccinimidylpropionate)] (Thermo Fisher Scientific; Rockford, IL) was added at 4°C for additional 2 h. Cells were spun down and lysed. Antibodies recognizing gH (Virostat Inc., Portland, ME), pUL130, β1 (Millipore), β3 integrins (Millipore) or an IgG isotype control (Santa Cruz Biochenology, Inc.) were added to lysate overnight at 4°C and then Protein A/G PLUS Agarose was added for 4 h at 4°C. Protein A/G PLUS Agarose beads with bound protein complexes were spun down, washed with a lysis buffer and resuspended in Laemmli Sample Buffer (BioRad Laboratories) containing β-mercaptoethanol (Fisher Scientific). Proteins were separated on SDS-PAGE gels, and transferred to ImmunoBlot PVDF membranes. Antibodies recognizing the HCMV gH (Virostat Inc.), pUL130, β1 or β3 integrins (Santa Cruz Biotechnology, Inc.) were used in the western blot assays.

### Quantitative and semiquantitative reverse transcriptase-PCR

Monocytes or fibroblasts were pretreated and infected. Total cellular DNA was harvested with E.Z.N.A. Tissue DNA Kit (Omega Bio-Tek, Inc.). The quantitative PCR amplification and detection were performed as previously described [27]. Semiquantitative PCR was also used to assess the entry efficiency of different HCMV strains and the efficiency of dynamin knockdown. Product amplification was carried out using MyCycler thermocycler (BioRad Laboratories), with the following PCR mix: 1× iTaq buffer (BioRad Laboratories) containing 1.25 U of iTaq DNA polymerase (Invitrogen Corp.) and a 50 µM concentration of each deoxynucleotide (Invitrogen Corp.). Primers specific for HCMV IE1-72 (sense, 5′-AGTGACCGAGGATTGCAACG-3′; antisense, 5′-CCTTGATTCTATGCCGCACC-3′), GAPDH (sense, 5′-GAAGGTGAAGGTGGAGT-3′; antisense, 5′-GAAGATGGTGATGGGATTTC-3′), 18S rRNA (sense, 5′-CGAGCCGCCTGGATACC-3_; antisense, 5′-CAGTTCCGAAAACCAACAAAATAG-3′) and dynamin (Santa Cruz Biotechnolgy, Inc.) were used to amplify target sequences. All primers, exempt primers for dynamin, were obtained from Integrated DNA Technologies, Inc. (Coralville, IA).

### siRNA transfection

Monocytes or fibroblasts were resuspended in Human Monocyte Nucleofector Solution or Basic Nucleofector Solution - Primary Fibroblasts, respectively (Lonza Group Ltd, Basel, Switzerland) containing 300 nM of paxillin siRNA (Dharmacon, Inc., Lafayette, CO; sequence: 5′-GUGUGGAGCCUUCUUUGGUUU-3′), 300 nM of dynamin siRNA (Santa Cruz Biotechnology, Inc.; sequence: 5′-CCAUCAUGCACCUCAUGAUTT), 300 nM control siRNA (Santa Cruz Biotechnology, Inc.) or RNase-free water (Invitrogen Corp.) and then transfected using an AMAXA Nucleofector (Lonza Group Ltd). siRNA-transfected monocytes or fibroblasts were mixed with pre-equilibrated Human Monocyte Nucleofector Medium [supplemented with 10% human serum (Sigma-Aldrich Inc.)] or Basic Nucleofector Medium – Primary Fibroblasts [supplemented with 4% fetal bovine serum (Sigma-Aldrich Inc.)], respectively (Lonza Group Ltd) and incubated for 48 h at 37°C in 5% CO_2_.

### HCMV entry assay

Performed as previously described [26], [27]. Briefly, monocytes, fibroblasts, or epithelial cells were treated and then HCMV infected (M.O.I. of 0.1) for 1 h at 4°C, washed with 1× PBS (Mediatech, Inc.) and temperature shifted to 37°C for 1 h. Cells were washed and treated with 2 mg/ml solution of Proteinase K (Promega) for 1 h at 4°C. Monocytes or fibroblasts were then harvested, total DNA was isolated and quantitative real-time PCR or semiquantitative PCR were performed using primers complementary to genomic HCMV DNA (the UL123/IE1-72 Exon 1 region) and cellular 18S rRNA. Results were plotted as a mean ±SEM. Student's T-tests were performed and *p* value of <0.05 was used for the measurement of statistical significance between samples. To monitor the accuracy of the assay, representative samples of infected monocytes were kept at 4°C without a temperature shift and processed as described above.

### Measurement of a functional HCMV entry

Isolated monocytes were infected with BAD*wt* or BAD*r*UL131 (MOI = 1) at 37°C for 1 hour then plated on fibronectin-coated cell culture dishes in RPMI 1640 supplemented with 10% human serum. Cells were incubated at 37°C in 5% CO_2_ for 5 days and 3 weeks post infection and media was changed every 5 days. At 5 days post infection, total DNA was extracted. At 3 weeks post infection, total RNA was extracted. Semiquantitative PCR and RT-PCR were performed using primers complementary to the viral UL123/IE1-72 Exon 1 region and cellular 18S rRNA.

### Fluorescence *in situ* hybridization

Alexa Fluor 488-labelled DNA probe was synthesized according to a protocol in FISH Tag DNA Green Kit (Invitrogen) using BAC DNA encoding the whole HCMV genome. Monocytes were HCMV infected (M.O.I. of 5) and kept at 37°C in 5% CO_2_ in RPMI 1640 supplemented with 10% human serum for 5 days. Cells were washed twice with 1× PBS and then fixed for 15 min. with 4% paraformaldehyde (Fisher Scientific). Cells were cytospun onto slides, dried and fixed with methanol (VWR, International, Radnor, PA):acetic acid (VWR International) (3∶1) for 15 min at room temperature (RT). Then cells were dehydrated at RT by subsequent washes with 70%, 85%, and 100% ethanol (Alcohol and Chemicals Co., Shelbyville, KY). Next, DNA inside cells was denatured by incubating cells with 70% formamide (Fisher Scientific) in 2× SSC (Boston BioProducts) at 70°C for 2 min. The dehydratation step followed at −20°C with subsequent washes with 70%, 80%, and 95% ethanol. Denatured DNA probe was then applied to cell spots and hybridization continued overnight at 37°C. After this time, cells were washed with 50% formamide in 2× SSC at 37°C, washed in 1× PBS and the blocking solution [1× PBS/5% normal goat serum (Santa Cruz Biotechnology, Inc.)/1∶80 of Fc Blocking Reagent (Miltenyi Biotec GmbH, Bergisch Gladbach, Germany)/0.3% Triton X-100 (Fisher Scientific)] was applied for 1 h at RT. Next, cells were incubated overnight with 20 µg/ml of anti-Alexa 488 rabbit IgG (Invitrogen) resuspended in the blocking solution. After this time, cells were washed with 1× PBS, the blocking solution was applied for 1 h. Cells then were incubated for 1 h with goat anti-rabbit Alexa 647 IgG (Invitrogen) resuspended in the blocking buffer. Next, cells were washed and mounted using SlowFade Gold with DAPI antifade reagent (Invitrogen). Images were taken using Leica TCS SP5 confocal microscope.

## Supporting Information

Figure S1 **Characterization of BADwt and BADrUL131, and their binding to monocytes and fibroblasts.** (A) Approximately 2×10^6^ virions of BADwt, BADrUL131, AD169 and TB40/E were spun down through a sucrose cushion, lysed and western blot analyses were performed using antibodies recognizing the HCMV proteins, pp65 and pUL130. (B). Monocytes were mock- or HCMV (BAD*wt* or BAD*r*UL131)-infected (M.O.I. of 5) and harvested at 15 min. pi. Densitometry analysis was performed based on western blot experiments where antibodies specific for the phosphorylated and non-phosphorylated forms of Src, and total actin were used. Monocytes (C) and fibroblasts (D) were infected (M.O.I. of 0.1) with BADwt or BADrUL131 for 1 h at 4°C. Cells were washed and harvested. qPCR was performed using primers complementary to genomic HCMV DNA and 18S rRNA. Results are plotted as a mean ±SEM. Student's T-tests were performed and p<0.05 was used for the measurement of statistical significance between samples. The experiments were repeated at least three times.
(TIF)

Figure S2 **HCMV entry at 4°C and 37°C, the role of integrins in the ability of BAD*wt* and BAD*r*UL131 to trigger Src/paxillin-signaling in monocytes, paxillin knockdown in monocytes and fibroblasts, and the effect of α-Thrombin on paxillin activation.** (A) Monocytes were infected with BADwt, BADrUL131, TB40/F, and TB40/E (M.O.I. of 0.1) for 1 h at 4°C, then left at 4°C or temperature shifted to 37°C for 1 h. Next, monocytes were washed and treated with Proteinase K solution for 1 h. Cells were then harvested and semiquantitative PCR was performed using primers complementary to genomic HCMV DNA and 18S rRNA, as an internal control. PCR reactions were analyzed by agarose gel electrophoresis using ethidium bromide. (B and C) Cells were untreated or pretreated with 5 µg/ml of blocking anti-β1 or anti-β3 integrin antibodies. Monocytes were then mock- or HCMV (BAD*wt* or BAD*r*UL131)-infected (M.O.I. of 5) and harvested at 15 min. pi. Monocytes (D) and fibroblasts (F) were transfected with siRNA complementary to paxillin or control siRNA for 48 h. Cells were then harvested. (E) Monocytes were mock-treated or treated with 5 U/ml α-thrombin and harvested at 15 min. pi. (B, C, D, E, and F) Western blot analyses were performed using antibodies specific for the phosphorylated and non-phosphorylated forms of Src and paxillin. Actin was used as a loading control.
(TIF)

Figure S3 **HCMV rearranges the actin cytoskeleton and regulates dynamin to efficiently enter into monocytes and epithelial cells. ** (A) Monocytes were isolated and then mock- or HCMV (Towne p.40)-infected (M.O.I. of 5). Monocytes were harvested at the time points indicated. (B) Monocytes were treated with DMSO, 0.5 µM jasplakinolide or 2.5 µM latrunculin A for 15 min. at 37°C/5% CO_2_ and then cells were harvested. (C) Monocytes were transfected with siRNA complementary to paxillin mRNA or a control siRNA for 48 h and then cells were harvested. (A, B, and C) Western blot analyses were performed using antibodies specific for F-actin and total actin. The experiment was repeated at least three times and the results are depicted as a ratio of F-actin to total actin measured by densitometry analysis (* represents statistical significance). (D) Monocytes were pretreated with DMSO, 50 µg/ml nystatin, 200 µM genistein, 100 µM Rac1 inhibitor or 50 µM dynasore. (F) HMECs were pretreated with 1 µM PP2, 1 µM AG1478, 0.5 µM jasplakinolide, 2.5 µM latrunculin A, or 50 µM dynasore. (D and F) Then, cells were HCMV (BADrUL131 or TB40/E)-infected at M.O.I. of 0.1 for 1 h at 4°C and next temperature shifted to 37°C for 1 h. Cells were washed and treated with Proteinase K solution for 1 h. Cells were then harvested and semiquantitative or real time-PCR analyses were performed using primers complementary to genomic HCMV DNA and 18S rRNA. Results are plotted as a mean ±SEM. Student's T-tests were performed and p<0.05 (indicated by asterisks) was used for the measurement of statistical significance between samples. The experiments were repeated at least three times. (E) Monocytes were transfected with siRNA complementary to dynamin mRNA or a control siRNA for 48 h. After this time, cells were harvested and semiquantitative PCR analysis was performed using primers complementary to dynamin mRNA or 18S rRNA. Reverse transcriptase negative (RT-) sample was used as a control.
(TIF)
