# Supplementary figures and images for: The HCMV gH/gL/UL128-131 Complex Triggers the Specific Cellular Activation Required for Efficient Viral Internalization into Target Monocytes
Source: PLoS Pathog. 2013 Jul 11;9(7):e1003463. doi: 10.1371/journal.ppat.1003463 (PMC3708883; doi:10.1371/journal.ppat.1003463)

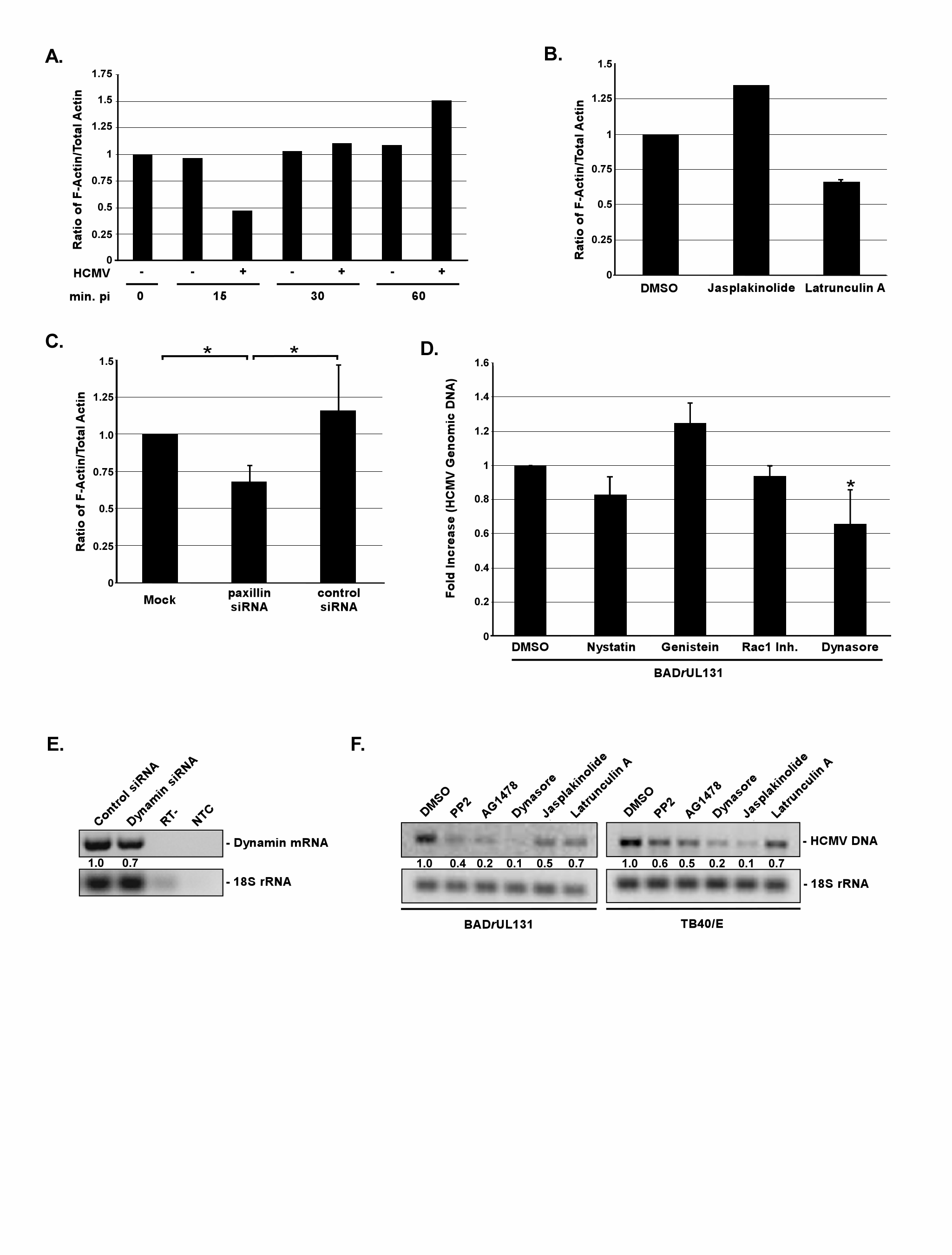

Supplement: Figure S3 — HCMV rearranges the actin cytoskeleton and regulates dynamin to efficiently enter into monocytes and epithelial cells. (A) Monocytes were isolated and then mock- or HCMV (Towne p.40)-infected (M.O.I. of 5). Monocytes were harvested at the time points indicated. (B) Monocytes were treated with DMSO, 0.5 µM jasplakinolide or 2.5 µM latrunculin A for 15 min. at 37°C/5% CO2 and then cells were harvested. (C) Monocytes were transfected with siRNA complementary to paxillin mRNA or a control siRNA for 48 h and then cells were harvested. (A, B, and C) Western blot analyses were performed using antibodies specific for F-actin and total actin. The experiment was repeated at least three times and the results are depicted as a ratio of F-actin to total actin measured by densitometry analysis (* represents statistical significance). (D) Monocytes were pretreated with DMSO, 50 µg/ml nystatin, 200 µM genistein, 100 µM Rac1 inhibitor or 50 µM dynasore. (F) HMECs were pretreated with 1 µM PP2, 1 µM AG1478, 0.5 µM jasplakinolide, 2.5 µM latrunculin A, or 50 µM dynasore. (D and F) Then, cells were HCMV (BADrUL131 or TB40/E)-infected at M.O.I. of 0.1 for 1 h at 4°C and next temperature shifted to 37°C for 1 h. Cells were washed and treated with Proteinase K solution for 1 h. Cells were then harvested and semiquantitative or real time-PCR analyses were performed using primers complementary to genomic HCMV DNA and 18S rRNA. Results are plotted as a mean ±SEM. Student's T-tests were performed and p<0.05 (indicated by asterisks) was used for the measurement of statistical significance between samples. The experiments were repeated at least three times. (E) Monocytes were transfected with siRNA complementary to dynamin mRNA or a control siRNA for 48 h. After this time, cells were harvested and semiquantitative PCR analysis was performed using primers complementary to dynamin mRNA or 18S rRNA. Reverse transcriptase negative (RT-) sample was used as a control. (TIF) [file ppat.1003463.s003.tif]
